# All‐in‐One, Wireless, Stretchable Hybrid Electronics for Smart, Connected, and Ambulatory Physiological Monitoring

**DOI:** 10.1002/advs.201900939

**Published:** 2019-07-24

**Authors:** Yun‐Soung Kim, Musa Mahmood, Yongkuk Lee, Nam Kyun Kim, Shinjae Kwon, Robert Herbert, Donghyun Kim, Hee Cheol Cho, Woon‐Hong Yeo

**Affiliations:** ^1^ George W. Woodruff School of Mechanical Engineering Institute for Electronics and Nanotechnology Georgia Institute of Technology Atlanta GA 30332 USA; ^2^ Department of Biomedical Engineering Wichita State University Wichita KS 67260 USA; ^3^ Department of Pediatrics School of Medicine Emory University Atlanta GA 30322 USA; ^4^ Department of Pediatrics Yonsei University College of Medicine Seoul 03722 South Korea; ^5^ Department of Surgery Yonsei University Wonju College of Medicine Wonju Gangwon‐do 220701 South Korea; ^6^ Wallace H. Coulter Department of Biomedical Engineering Parker H. Petit Institute for Bioengineering and Biosciences Georgia Institute of Technology and Emory University Atlanta GA 30332 USA; ^7^ Center for Flexible and Wearable Electronics Advanced Research Institute for Materials Neural Engineering Center Georgia Institute of Technology Atlanta GA 30332 USA

**Keywords:** ambulatory cardiac monitoring, physiological signals, stretchable hybrid electronics, wearable electronics

## Abstract

Commercially available health monitors rely on rigid electronic housing coupled with aggressive adhesives and conductive gels, causing discomfort and inducing skin damage. Also, research‐level skin‐wearable devices, while excelling in some aspects, fall short as concept‐only presentations due to the fundamental challenges of active wireless communication and integration as a single device platform. Here, an all‐in‐one, wireless, stretchable hybrid electronics with key capabilities for real‐time physiological monitoring, automatic detection of signal abnormality via deep‐learning, and a long‐range wireless connectivity (up to 15 m) is introduced. The strategic integration of thin‐film electronic layers with hyperelastic elastomers allows the overall device to adhere and deform naturally with the human body while maintaining the functionalities of the on‐board electronics. The stretchable electrodes with optimized structures for intimate skin contact are capable of generating clinical‐grade electrocardiograms and accurate analysis of heart and respiratory rates while the motion sensor assesses physical activities. Implementation of convolutional neural networks for real‐time physiological classifications demonstrates the feasibility of multifaceted analysis with a high clinical relevance. Finally, in vivo demonstrations with animals and human subjects in various scenarios reveal the versatility of the device as both a health monitor and a viable research tool.

## 1 Introduction

Monitoring the electrocardiogram (ECG) is one of the greatest interests to both healthcare providers and patients alike since it manifests the heart's condition with a relatively simple setup. Many cardiac abnormalities such as myocardial ischemia/infarction and several types of arrhythmia can be detected by inspecting the waveforms collected by ECG.^1^, ^2^ ECG also provides clinicians a range of indirect measures of the patient's condition including prognostic estimation, postoperative recovery, drug efficacy, mental stress, and risk of sudden cardiac death.^3^, ^4^, ^5^, ^6^, ^7^, ^8^, ^9^ For the latter type of investigations, healthcare providers need to assess the heart for extended periods and evaluate the trends in long‐term cardiac activities to make appropriate clinical decisions. In these scenarios, the standard method for diagnosis is a Holter monitor, a portable ECG device that can be strapped to the patient for a 24 h ambulatory ECG recording. Despite its value in the acquisition of long‐term ECG data, Holter monitors have limitations as the following: 1) the entire set up, which includes the data acquisition unit, bundles of cables, gelled adhesives electrodes, tapes and a strap, obstruct the natural movements of the patient's body and create extreme discomfort which disrupt daily activities and sleep, 2) gels can dry over time and degrade the signal qualities, 3) gels and adhesives can cause skin irritation, 4) the user cannot shower, adding to the overall discomfort, 5) since the equipment needs to be returned to the clinic for data transfer and analysis, it requires the patient to plan additional visits, and 6) clinicians will be observing cardiac events of the past: it is impossible to perform a timely intervention when unexpected cardiac events occur. Consequently, there is a high demand for a cardiac monitoring solution that provides not only the acquisition of clinical‐grade ECG, but also the form factor non‐disruptive to everyday tasks as well as the real‐time, continuous analysis of the patient's ECG for both improved diagnosis and timely intervention. Numerous commercially available wearable ECG monitors have been presented; however, these devices lack the mechanical compliance for skin and induce physical stresses to the application site due to the rigid construction of the electronics.^10^ Furthermore, the application of the devices relies on aggressive acrylic‐based adhesives and exposes the patients to potential tissues damages during the removal process—key disadvantage that restricts the use of technology for neonate or young children who have fragile skin conditions.^11^ While several research studies attempted to address above‐mentioned issues associated with commercial wearable cardiac monitors, the presented devices fall short as feasible solutions due to the nature of “sensor‐only” presentation, incorporation of a rigid printed circuit board, short‐range wireless connectivity, or use of a conductive gel,^12^, ^13^, ^14^, ^15^, ^16^, ^17^, ^18^ the use of which is not desirable due to potential skin irritation and drying over long‐term recordings. Here we present the first demonstration of a wireless, “all‐in‐one” stretchable hybrid electronic system (SHE) that can monitor the user's real‐time cardiac conditions through the continuous assessment of the recorded ECG as well as the detection and notification of clinically significant cardiac events. ECG‐derived heart rate (HR) and respiratory rate (RR) along with the onboard motion sensor provide valuable and critical set of physiological information without compromising device's formfactor. The hyperelastic construction allows the device to deform naturally with user's movement while supplying sufficient adhesive forces to adhere the device throughout daily activities and strenuous exercises. Since a skin‐conformal, low‐modulus elastomer has shown negligible skin irritation,^19^, ^20^ three gold ECG electrodes are integrated with the elastomer substrate to allow the formation of a gapless electrode‐to‐skin interface and obviate the need for the use of conductive gels. The hybrid integration of miniature electronic packages with an ultrathin interconnection platform allows the on‐board implementation of advanced signal processing and a long‐range Bluetooth connectivity. The low‐modulus elastomer constitutes the majority of SHE, allowing the device to impart overall stretchability despite the presence of non‐stretchable components, such as the interconnection and chips. Continuous wireless connectivity with increasingly powerful smartphones and tablets allow for deployment of deep learning solutions, such as convolutional neural networks (CNNs), for real‐time analysis of patient conditions that are readily accessible. In this study, implementation of two CNNs incorporating *inception*‐type convolutional units with residual connections are demonstrated for classifying user activity from acceleration and angular velocity,^21^ and semantic segmentation of ECG ectopic beats and arrhythmias for cardiac conditions.^22^ To determine accuracy, two publicly available ECG datasets, the PTB Diagnostic ECG Database and the St. Petersburg Institute of Cardiological Technics 12‐lead (INCART) Arrhythmia Database, are used to evaluate the machine‐learning model using fivefold cross‐validation.^23^ Finally, feasibility of the device for diverse clinical and research scenarios are investigated by conducting the treadmill exercises, prolonged application exceeding one week, and animal study involving moving rats.

## 2 Results

### 2.1 Overview of the System Architecture and Functionalities of Ambulatory Health Monitoring

**Figure 1:**
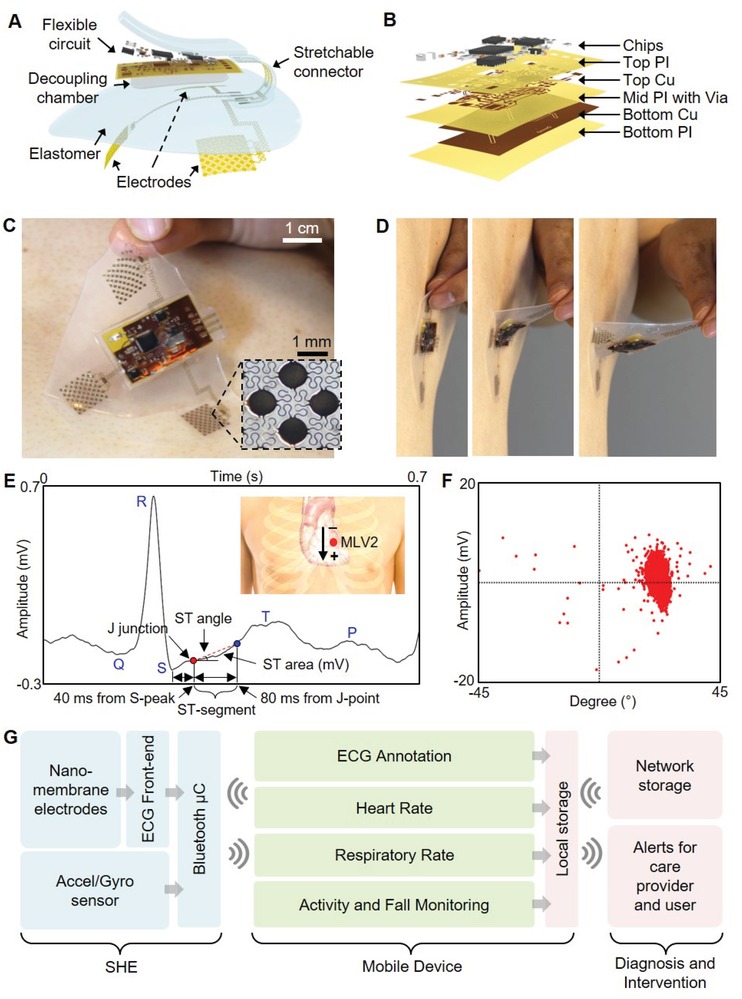
Overview of the SHE and data management. A) Schematic illustration showing the main structural components and their assembly for SHE. B) Blow‐up rendering image detailing the layer information of the flexible circuit. C) Photo of an SHE showing the soft and adhesive property for direct lamination with the skin without the use of adhesives. D) Side view of delamination sequence of the ultrathin device with no negative effects to the skin. E) Detail inspection of ECG acquired by the SHE in (C) and identification of PQRST waves, J‐point, and ST‐segment angle. Inset diagram describes the position of SHE (red dot) and polarity of the two measurement electrodes. F) Scatter plot showing the angle and amplitude distribution of ST‐segments of the ECG data in (E). G) Flow‐chart illustration of a real‐time, smart, and ambulatory health monitoring, enabled by the SHE.

**Figure** **1**A illustrates the main structural and functional components that make up the wireless, stretchable hybrid electronics (SHE), while Figure 1B describes the layer‐by‐layer structure of the on‐board flexible electronics. The assembly process consists of four phases: 1) fabrication of the thin‐film circuit and nanomembrane electrodes, 2) transfer of the thin‐film structures to elastomeric substrates, 3) circuit assembly by reflow soldering of the surface mount components, and 4) final packaging including electrode connection, elastomer encapsulation, creation of the oil chamber, and magnet attachment for the battery connection. We utilized a set of standard microfabrication steps to construct the thin, flexible, and stretchable polymer/metal composite structures and to laminate onto an elastomeric substrate. Conventional reflow soldering using a solder paste (SMDLTLFP10T5, Chip Quik) and surface mount chip components were used for circuit assembly, following previously reported methods.^24^, ^25^ Details of the fabrication process are described in the Supporting Information (Figure S1 and Note S1), and the description of the circuit design and the chip components can be found in the Supporting Information (Figure S2 and Table S1). Since the major structural component that comprises the SHE is prepared by casting a mixture of elastomers with extremely low moduli, the device is naturally adhesive to the skin and compliant for stretching and bending (Figure 1C,D). The integrated circuit components are fully concealed and isolated by the elastomer and are powered by an external power source, such as the miniature lithium‐ion poly‐mer battery (DTP301120, Shenzhen Data Power Technology). Supporting Information (Figure S3) details the powering of the SHE using the magnetic electrical interfaces, the power efficiency of the device, and waterproofing strategy for continuous monitoring even in the presence of water. The set of three nanomembrane gold electrodes that contact the skin are patterned photolithographically into a network of repeating units of circular islands bridged with meander lines (inset photo in Figure 1C), allowing for both stretchability and conformal contact to the skin. The electrodes are configured in a bipolar setup and are intended to mimic precordial (transverse plane) lead V2, which we refer to as Modified Lead V2 (MLV2). From experimentation, we determined that the bipolar surface electrodes produce an accurate V2 analog lead. To verify the quality of ECG acquired by the SHE, we inspected the collected waveform in detail as shown in Figure 1E,F. As the results show, the SHE is fully capable of capturing the details of the human ECG including all of P wave, T wave, QRS complex, ST‐segment, and J‐point, the dynamics of which are useful markers associated with asymptomatic ischemia.^26^, ^27^ The acquired ECGs were also compatible with clinically relevant analysis, such as the R‐R variation, low‐frequency band (LF)/high‐frequency band (HF) ratio, pNN50 (time domain measure of heart rate variability), and SD1/SD2 (standard deviation ratio from heart rate variability), confirming the SHE's performance in clinically relevant ECG acquisition (Figure S4, Supporting Information). The flowchart diagram in Figure 1G describes the overview of the method with which SHE is utilized for continuous patient monitoring. The raw physiological data are transmitted via Bluetooth to the connected mobile Android device, where they are processed through the CNN‐based deep‐learning algorithms and classified into subcategories in real time. Further, the ECG is simultaneously conditioned using R‐peak detection and interpolation algorithms to determine the user's HR (beats min^−1^) and RR (breaths min^−1^). These four datasets are continuously displayed on the mobile device for real‐time user inspection and stored on the device's local storage for future analysis. Finally, the data can be synced to a network accessible to the healthcare provider for real‐time assessment of the patient and timely response for emergency events, such as a heart attack. A detail example of the graphical user interface of the Android application is shown in the Supporting Information (Figure S5). A human body has significant effects on the radiation characteristics of the antenna due to the tissue's scattering and absorption of the electromagnetic waves. Since the SHE is designed to integrate intimately with the human body, an effort was made to ensure the long‐range connectivity of the BLE by incorporating a T‐matching network with experimentally determined passive components to maximize the RF efficiency at 2.45 GHz despite the extreme proximity to the skin (Figure S6, Supporting Information). As a result, the SHE maintained the wireless connectivity up to 15 m while operating on a user, confirming the tether‐free characteristic of the device (real‐time demonstration in Movie S1, Supporting Information).

### 2.2 Optimization of the Elastomeric Properties for Improved Signal Qualities

**Figure 2:**
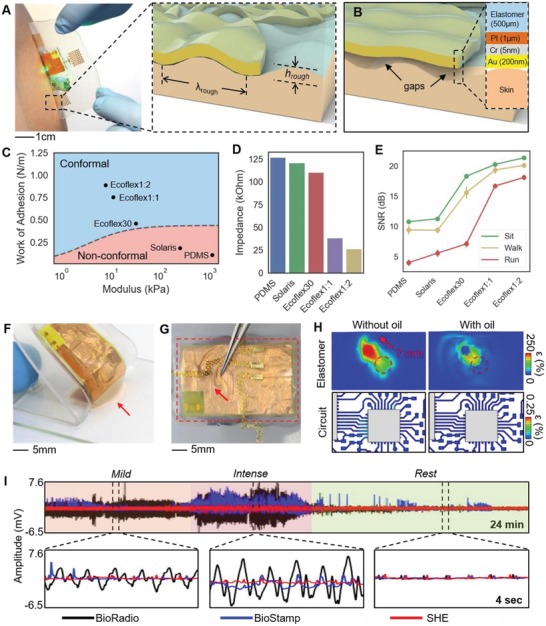
Mechanics and materials for enhanced physiological monitoring. A) Photo of the SHE lamination to the skin. The inset shows interfacial mechanics between skin and electrode during conformal contact. B) Illustration showing a nonconformal contact with the presence of gaps. The inset details the layer information. C) Determination of conformal contact: dotted curve represents the critical contact points of 500 µm thick elastomers. D) Skin‐electrode contact impedance with five different elastomers. E) SNR of ECG collected with a subject during idle, walk, and run. F) Photo of showing the attribute of the oil‐filled decoupling chamber with the bottom elastomer (red arrow) trailing the movement of the top circuit. G) Photo of capturing the effect of the decoupling chamber when a tweezer pushes the electrode layer. H) Computation of maximum principal strains induced on the Cu interconnects by linearly displacing a circular portion of electrode elastomer by 7 mm. Maximum strain in Cu is 250% and 0.25% for the absence and presence of the decoupling chamber, respectively. I) Comparison of ECG measured by two commercial devices (BioRadio and BioStamp) and SHE. SHE shows negligible motion artifacts.

One of the key advantages of the SHE is that it obviates the need for application of conductive gels and adhesives. The extremely thin Au/PI electrodes (thickness: 1.2 µm) with the stretchable mesh layout can “hug” the complex skin surface structures while being able to freely deform with the dynamic movements of the tissue. While prior studies investigated the effects of reducing the membrane thickness with aims of providing conformal contact with the skin,^12^, ^28^, ^29^ these results lacked the translation of the findings into practical applications—extremely thin membranes are not only fragile to retrieve from the skin but also are challenging to interface with rigid electronic packages. Here, we present a novel construction strategy for wearable electronics that preserves both the seamless electrode‐to‐skin contact and the means to interconnect the thin‐film electrodes with the on‐board integrated circuit. A 500 µm thick elastomer was chosen to serve as the main substrate that helps maintain the electrodes' integrity during the application and retrieval of the device (**Figure** **2**A). While the increased substrate thickness ensures the electrode integrity during device handling and retrieval, the added thickness could influence the level of conformal contact, which can have direct consequences in the qualities of ECG. Since conformal contact of the electrodes is dependent on design and material properties, an analytical model of the interfacial mechanics is applied to understand the criteria for conformal contact using the variables that represent skin's roughness (λ_rough_ and *h*_rough_, Figure 2A inset) and layer information provided in Figure 2B. Conformal contact occurs when adhesion energy is greater than the sum of the skin's elastic energy and electrode's bending energy.^30^ Adhesion energy is based on the elastomer's work of adhesion (γ_elastomer_) and exposed surface area. The elastic energy depends on the modulus of skin (*E*_skin_) and λ_rough_. Bending energy is determined by modeling the electrode as a composite material of Au, Cr, PI, and elastomer, contributing to an effective bending stiffness (EI). Here, we investigate the relationship between γ_elastomer_ and modulus (*E*_elastomer_) necessary to achieve conformal contact of the electrode. We assume the on‐board electronics are mechanically independent of the underlying electrodes and interconnects due to the thick, soft elastomer layer. Electrode design and layer thicknesses are held constant. For a given *E*_elastomer_, there is a minimum γ_elastomer_ to provide conformal contact. This critical adhesion value is expressed by

(1) $$\gamma_{\text{elastomer}}\;\;>\;\;\left(\frac{1}{1-\alpha }\right)\;\;\frac{\frac{\pi^{4}EIh^{2}}{\lambda_{\text{rough}}^{4}}\;\;+\;\;\frac{\pi E_{\text{skin}}\left(h_{\text{rough}}\;\;-\;\;h\right)}{16\lambda_{\text{rough}}}}{\left(1\;\;+\;\;\frac{\pi^{2}h^{2}}{4\lambda_{\text{rough}}^{2}}\right)}$$

where $h\;\;=\;\;\frac{E_{\text{skin}}h_{\text{rough}}}{\frac{16\pi^{3}EI}{\lambda_{\text{rough}}^{3}}\;\;+\;\;E_{\text{skin}}}$ and α is the areal fraction of PI and Au.

The dotted boundary curve shown in Figure 2C represents the critical points and indicates the 500 µm thick elastomer properties required for conformal contact. Details of the analysis process leading up to expression (1) are described in the Supporting Information (Note S2). To examine the validity of the model, four different silicone elastomer products were used: 1) polydimethylsiloxane (PDMS, Dow Sylgard 184) with a base‐to‐cure ratio of 10:1, 2) Solaris (Smooth‐On), 3) Ecoflex 0030 (Smooth‐On), and 4) Ecoflex GEL (Smooth‐On). Since Ecoflex GEL's extremely low modulus and tackiness prevented forming a usable layer, we modulated its properties by adding Ecoflex 0030 with two different weight ratios. First modification was prepared by adding an equal part of Ecoflex GEL to Ecoflex 0030 (referred to as “Ecoflex1:1”) and the second modification was prepared by adding two parts of Ecoflex GEL to Ecoflex 0030 (referred to as “Ecoflex1:2”). This method allowed the formulation of five different candidate elastomer substrates with varying degrees in the elastomer properties. Tensile stress tests using a digital force gauge (M5‐5, Mark‐10) fixed on a motorized test stand (ESM303, Mark‐10) allowed us to measure the modulus of each material. The measured moduli for PDMS (1131.04 kPa), Solaris (251.53 kPa), and Ecoflex 0030 (32.23 kPa) were within the range of previously reported values with possible deviation arising from different mixing/curing conditions and the Poisson's effect.^28^, ^31^, ^32^ The moduli for Ecoflex1:1 and 1:2 hybrids were measured to be 11.17 and 7.85 kPa, respectively (Figure S7, Supporting Information). Experiments to obtain the work of adhesion for each sample were conducted by rolling metal cylinders down a track coated with each elastomer and observing the effective changes in the peel energy (∆γ) at various speeds.^33^ We varied the rolling speeds by using five cylinders with different dimensions and varying the track's angles of incline (5°, 10°, 20°, and 30°). The set of data points for each elastomer allowed us to determine the work of adhesion by finding the y‐intercept of the curve fit line. Details of the experimental setup and results can be found in the Supporting Information (Figures S8 and S9 and Table S2). Using the experimentally obtained *E*_elastomer_ and γ_elastomer_, the conformality criteria for each elastomer could be determined. As shown in Figure 2C, PDMS and Solaris were categorized as *non‐conformal*, Ecoflex 0030 as *conformal* with a small margin, and Ecoflex1:1 and 1:2 as *conformal* with a large margin. Non‐conformality of the elastomer and electrodes results in decreased effective electrode area and is expected to reduce the amplitudes of the acquired ECG. Moreover, the gaps formed between the skin and metal film electrodes can deteriorate the signal qualities by introducing significant motion artifacts due to the movement‐induced changes in the resistance and capacitance properties of the interface. In order to confirm the influence of elastomer properties on electrode performances, we prepared five sets of electrodes with each integrated on a different elastomer and measured the electrode‐to‐skin contact impedance (*Z*_c_), a low quantity of which is desired in acquisition of low frequency biopotentials, such as ECG. Minimizing *Z*_c_ also helps maximize the differential amplifier's ability to attenuate the common noise present to both the active and reference electrodes, therefore increasing the signal‐to‐noise ratio (SNR).^34^ Detail explanation for SNR can be found in the Experimental Section. The measurement was done by placing the electrodes over a forearm, which had been cleansed with Scotch tapes (three times) and an alcohol wipe and connecting the positive and negative electrodes to a test equipment (Checktrode Model 1089 MK III, UFI). As Figure 2D reveals, contact impedance of the five sets of electrodes followed the same order found in *E*_elastomer_ and γ_elastomer_, with Ecoflex 1:2 and PDMS exhibiting the lowest and highest impedances, respectively. To examine the consequences of measured contact impedance and its influence on the acquired ECGs, we collected ECGs using the five electrodes while the subject was stationary, walking, and running, and compared the SNR. As described in Figure 2E, the average trend in SNR measured by the five elastomer electrodes followed the same order in *E*_elastomer_, γ_elastomer_, and *Z*_c_, with decreased SNR across all electrodes during walking and running. While electrodes on Ecoflex1:1 and 1:2 provided ECG with preserved features, all other electrodes suffered loss of features and significant baseline drifts, especially when the user was moving. Details of the ECG data can be seen in the Supporting Information (Figure S10).

While the conformal contact criteria for electrodes could be sufficiently achieved with Ecoflex1:2, portions of the SHE soldered with the surface mount chips would disrupt the compliant device mechanics, leading to poor device and electrode adhesion. In order to resolve this challenge, we designed a thin oil‐filled decoupling chamber under the circuit's footprint that separates the mechanics between the electrodes and electronics. The oil (Vegetable Glycerin, Essential Depot) was injected in the chamber using a micropipette and sealed with elastomer. The increase in the device adhesion could be observed when the device was peeled from a clean glass slide, with the elastomer directly below the chamber trailing the movement of the electronic layer (Figure 2F). Visually, the oil chamber could be seen to allow the electrode layer to slide freely with respect to the electronic layer (Figure 2G). We developed an ABAQUS model representing the electronic section of the SHE to quantify the effect of the decoupling chamber on the thin‐film Cu interconnects, whose mechanical integrity is directly related to device operation. The model simulated an in‐plane displacement (7 mm) of a circular region (diameter = 3.5 mm) of the electrode layer for both “with” and “without” a decoupling chamber. While 7 mm was chosen arbitrarily and is beyond the range of expected displacements during a normal use of the device, the maximum strain observed in the Cu interconnect for “with” scenario (ε = 0.00025%) was a thousandth of that of “without” scenario (ε = 0.25%), which approaches the yield strain of 0.5% for a 500 nm thick Cu film,^35^ (Figure 2H). This result indicates that the decoupling chamber helps prevent damages to the electronics not only during the normal application and removal of the device, but even from an accidental mishandling. The apparent increase in the adhesion of the device was further investigated by measuring the peel forces of the two “with” and “without” devices by peeling the devices off a forearm using a digital force gauge fixed on a motorized test stand. Integrating the “peel force versus displacement” curves of the two devices revealed that the total energy required to completely peel a device with a decoupling chamber (≈4.5 mJ) is more than twice the case without a decoupling chamber (≈2.1 mJ). Details of the experimental set up, description of the decoupling chamber, and the peel force plots can be found in the Supporting Information (Figure S11). Based on the results of the above analysis, we fabricated a complete SHE based on the Ecoflex1:2 elastomer and compared the ECG qualities to those acquired by commercially available devices, namely the BioRadio (Cleavmed Inc.) and BioStamp (MC10 Inc.). ECG was simultaneously recorded with SHE and BioRadio while the user performed a sequence of treadmill exercises with varying intensities. ECG with a BioStamp was recorded separately due to the overlap of the application site with the SHE. Photos of the device placement and orientation are show in the Supporting Information (Figure S12). As shown in Figure 2I, all three ECGs exhibited the similar quality while the user was resting. The BioRadio's data showed the most susceptibility to movement of any intensity, such as walking, and the artifacts buried the ECG, most likely due to the weights of the cables between the gel electrodes and the data acquisition unit. The BioStamp's data showed improved signal qualities, however still exhibited random spikes and baseline wandering likely due to the changes in the gel‐mediated electrode contacts. The SHE did suffer mild baseline wander during high intensity exercise, but displayed ECG with preserved features that can be used for further analysis.

The long‐term mechanical reliability of the SHE is tested by repeatedly exerting a metal cylinder (radius = 12.5 mm) against the circuit's footprint using a uniaxial servohydraulic testing machine (MTS Systems, Inc). SHE's continuous BLE connectivity to a nearby tablet was used as the indication for the electronics' integrity since any damage to the BLE, ADC, or voltage regulator would result in a loss of signal in the tablet's display. The SHE was displaced by the initial distance of 5.08 mm for 1000 cycles. After every 1000 cycles, we incremented the displacement by 2.54 mm, reaching the final displacement of 15.24 mm and a total of 5000 cycles. Maintaining BLE connectivity throughout the experiment illustrates the SHE's structural integrity as an electronic system despite the extreme case of repeated bending and stretching, whose criteria exceed the expected deformation during normal device applications. Details of the test setup and protocol are included in the Supporting Information (Figure S13 and Movie S2).

The long‐term reliability of the stretchable connector was investigated by a set of cyclic bending and stretching up to 1000 cycles, which is well beyond the expected strain range during the normal use of the SHE. The results in Figure S14 (Supporting Information) indicate that the stretchable connector maintained a stable resistance throughout 1000 cycles in both tests. The average variation in the resistance values were approximately 0.2% (0.5 Ω) and 0.4% (0.7 Ω) for cyclic stretching and bending, respectively. In addition, the long‐term performance of the complete device was inspected via a cyclic stretching; ECG was recorded after cyclically stretching the SHE up to 30% for total of 400 cycles. The average SNRs obtained from 10 min long ECG recorded from each cycle indicate that the SHE maintained the functionality. Moreover, there was no indication that the repeated stretching negatively affected the functionality of the device, evidenced by the SNR values of greater than 20 dB throughout the test. At any step, all ECG features, such as the P and T waves and the QRS complexes, could be sufficiently distinguished. Lastly, the maximum stretchability of the SHE was inspected by stretching the device until ECG could not be recorded. However, the SHE maintained reliable functionality even up to 60%, which is far beyond the extent of stretching even in accidental scenarios, and the test was concluded (Figure S15, Supporting Information).

### 2.3 Development of the Classification Algorithms for Real‐Time Monitoring of ECG, HR, RR, and Activity

**Figure 3:**
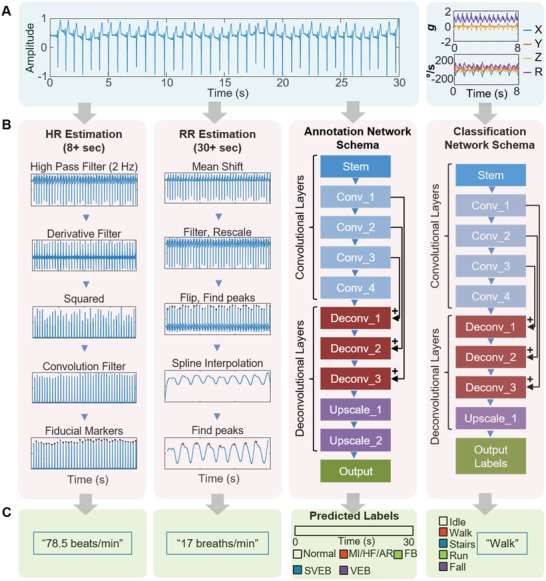
Development of CNN‐based algorithms for real‐time monitoring and classifications of four types of physiological signals. A) A set of ECG measured by an SHE (left) and motion activity, measured by change of acceleration (top‐right) and orientation (bottom‐right). B) Overview of data processing methods, separated into four columns: 1) HR estimation process, 2) RR estimation process, 3) ECG annotation via semantic segmentation network for detection of various types of cardiac disease (MI/HF/AR, FB, SVEB, and VEB), and 4) Classification network schema of motion activity data (idle, walk, stairs, run, and fall). C) Each column shows the respective outputs from the previous column in (B), in the order of HR, RR, ECG annotation, and motion activity class.

**Figure** **3** describes the entire data analysis process and development of CNN‐based algorithm for ECG, HR, RR, and motion activity data recorded by the SHE. The raw ECG, acceleration, and angular velocity data are simultaneously processed (Figure 3A) toward interpreting HR, RR, ECG annotation, and activity classification, where each process is represented as a separate column in Figure 3B. The corresponding outputs for each of the columns are shown in Figure 3C. Our ECG annotation model, named ECGSeq2Seq, uses a sequence‐to‐sequence annotation concept. The third column in Figure 3B shows a high‐level representation of ECGSeq2Seq, broken down into blocks, with the black arrows representing residual connections (filter concatenation) between prior *convolutional* layers, and corresponding *deconvolutional* layers of similar size. The detailed description showing the processing steps involved with the four outputs is shown in the Supporting Information (Figure S16). The ECG annotation model is detailed in Figure S17a (Supporting Information), along with the output dimensions from each layer. This is color coded to Figure S17b (Supporting Information), which details which individual layers belong under each block. The stem contains the initial steps performed by the model, involving dimensionality reductions through convolution operations and max pooling. Figure S17c (Supporting Information) shows the convolution parameters that are changed in the number of filters, filter size, and strides. It also shows the convolution layer is followed by a batch normalization layer^36^ and a linear activation. After the stem, we have four convolutional blocks, which are *Inception*‐type blocks from Szegedy et al.'s work on image classification with deep convolutional networks.^21^ The *deconvolutional* layers involve an up‐sampling step along the sequence length, by a factor of two, along with a convolution layer. Several residual connections are made between the convolutional layers, and corresponding deconvolutional layers. Residual connections are necessary to combat the vanishing gradient problem, which causes very deep networks to saturate then degrade in accuracy during training.^37^ The use of residual connections preserves gradient flow by bridging the gap between distant layers, improving training convergence and reducing overfitting.

This new ECGSeq2Seq network architecture uses an encoder‐decoder paradigm to perform semantic segmentation of ECG data, where each input data has corresponding output labels for each class. Long et al. used a similar method for pixel‐to‐pixel semantic segmentation of images.^22^ Here, the deconvolutional layers seek to use the features extracted in the first half of the model and, when combined with an accurate beat‐detection algorithm, is converted into a human‐understandable ECG annotation. The PTB dataset, included data from 290 unique subjects along with diagnosis information, and included multiple 12‐lead ECG recordings. The diagnosis information was used to label each of the datasets, separated into the following five categories (Figure 3C): 1) normal sinus rhythm (normal), 2) myocardial infarction (MI), heart failure (HF), and miscellaneous arrhythmia (AR), 3) fusion beat (FB), 4) supraventricular ectopic beats (SVEB), and 5) ventricular ectopic beats (VEB). This data set was combined with the INCART dataset, including 75 different recordings where each beat was individually labeled by a professional physician. This allows the model to be trained to recognize both ectopic beats as well as diagnose longer irregular rhythms. The PTB data were labeled after resampling from 1000 to 250 Hz and the INCART data were resampled from 257 to 250 Hz. As we intend to model annotation as a sequence‐to‐sequence model, each input data point from the 8 s, 2000‐point input was mapped to a 2000‐point sequence of labels spanning the classes. Therefore, the input shape is (2000, 1), and the output shape is (2000, 9), where 9 is the number of output classes. This differs from previous CNN‐based classification models in that it allows for precise beat labeling learned entirely from existing data. In order to train our ECGSeq2Seq annotation model, we calculate the loss as categorical cross‐entropy and use the Adam optimizer^38^ with a starting learning rate of 0.001. The data was separated into training and test sets using a 75–25% split, ensuring no overlap between the training and test set. This test was performed four times, in a four‐fold cross‐validation. The training set was further subdivided into the training and validation with an 80–20 split with no overlap. The total number of samples was 87 482 8 s windows at 250Hz. During the training process, the learning rate was reduced by a factor of 10 if there was no improvement to validation accuracy for consecutive epochs. The training and test accuracy appear in the Supporting Information (Figure S17d). A similar model was used for classification of activity from motion sensor data, as shown in the Supporting Information (Figure S18a–c). This model was named as ActivityResNet, due to the use of residual connections. Data was collected from five subjects using continuous monitoring of motion activity with the SHE on the chest. Each subject performed the following activities for 5 min each: idle sitting or standing, walking, walking upstairs, walking downstairs, and running.

Additionally, each subject was asked to fall five times while wearing the SHE for the fall detection portion of the training. The training process for this network was the same as the one for the ECGSeq2Seq model. The training and test accuracy are shown in Figure S18d (Supporting Information). The classification results for each of these models are presented as confusion matrices in Figure S19a,b (Supporting Information) for ECG and motion activity, respectively; the ECGSeq2Seq model achieved an average accuracy of 98.7 ± 1.4%, while the ActivityResNet model achieved 98.9 ± 3.3% over fourfold cross validation. Samples from each of the abnormal ECG classes are summarized in Figure S20 (Supporting Information).

### 2.4 In Vivo, Real‐Time Validation of the Designed Algorithms with Human Subjects

**Figure 4:**
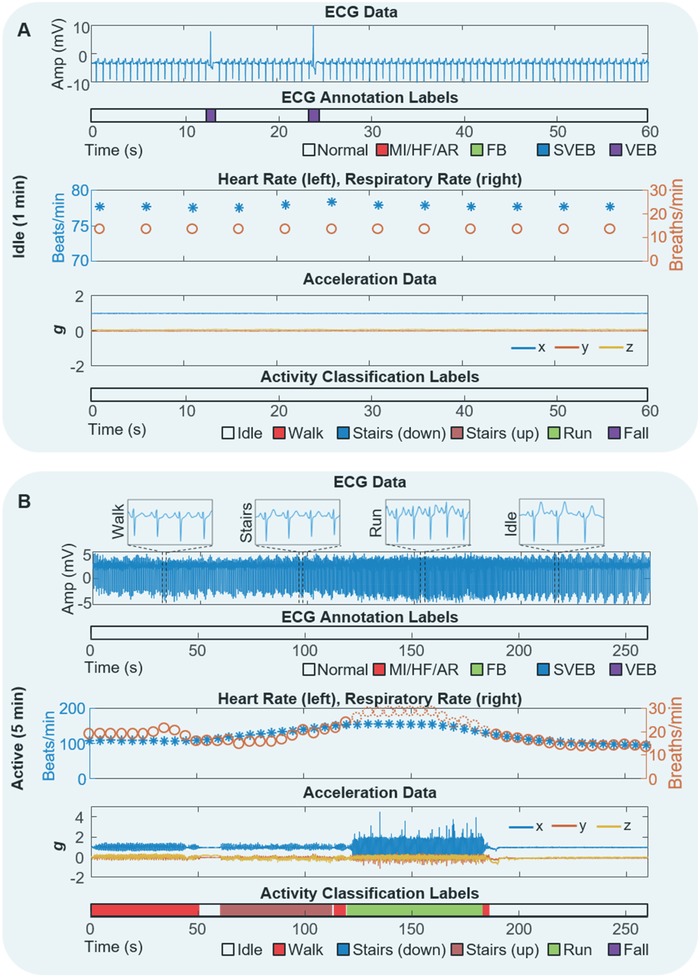
In vivo, real‐time validation of the designed algorithms with human subjects. A,B) A real‐time, ambulatory monitoring of ECG and motion activity with a SHE on a human subject. At both A) idle and B) active movements of a subject, the device clearly detects VEB in the annotation model. A variety of activities includes walking, climbing stairs, and running (B). The four zoom‐in plots in (B) show detail 2 s window of ECG waveforms from corresponding activity.

**Figure** **4** demonstrates multiple scenarios along with in vivo, real‐time analysis performed on a mobile device when using the SHE. This work validates the device performance on wireless, real‐time detection of ECG abnormality and motion activity. Initially, ECG is recorded as a baseline from a normal healthy subject during idle activity. The same device on another subject successfully detects a symptom of VEB from occasional premature ventricular contractions in real‐time (Figure 4A). Movie S3 (Supporting Information) shows real‐time monitoring of HR, RR estimation and ECG annotation on a tablet device for a healthy subject with no anomalous or ectopic ECG activity. We also demonstrate the real‐time detection and annotation of ectopic beats from an unhealthy subject (Movie S4, Supporting Information). As shown in Figure 4B, the SHE offers a wireless, portable, real‐time monitoring of ECG, HR, and RR during various activities, including walking, running, and walking upstairs. Any smartphone or tablet via Bluetooth connection can monitor the physiological signals in real time, while alerting any abnormality of ECG and emergency of falling (details appear in Movie S5, Supporting Information). To validate the device performance, we compare the SHE with a commercial system (BioRadio) in monitoring of ECG and respiratory inductance plethysmography (RIP). Both devices recorded signals simultaneously on a subject performing various activities (walking, running, and idle; Figure S19, Supporting Information). The corresponding set of ECG, derived HR, and RR for both devices is shown in Figure S21a–c (Supporting Information), respectively. The SHE‐derived HR is compared with the BioRadio data using a regression plot in the Supporting Information (Figure S18d) showing a high coherence with a slope of 0.9760, and a coefficient of determination of *R* ^2^ = 0.9844. Comparison of RR data (Figure S21e, Supporting Information) also shows a good agreement with a slope of 1.0545, and a coefficient of determination of *R* ^2^ = 0.9504. This result is quite significant considering the difficulty of deriving respiratory rate from a single ECG lead. Further tests were conducted to validate the SHE's accuracy in detecting RR. In this simulated scenario, both the SHE and BioRadio RIP simultaneously recorded an idle subject who increased the RR over time. The result shows that the SHE‐derived respiration curves closely represent that of the commercially available RIP device's respiration curves (Figure S22a,b, Supporting Information). Figure S22c (Supporting Information) shows the extended RR recording over time for 5 min with a high correspondence between the SHE and BioRadio. A regression plot comparing the two RR data (Figure S22d, Supporting Information) shows a coefficient of determination of *R* ^2^ = 0.9776. Overall, the SHE shows excellent performance when compared with the commercial hardware for monitoring representative physiological signals.

### 2.5 Demonstration of the SHE as a Versatile Clinical and Research Tool

**Figure 5:**
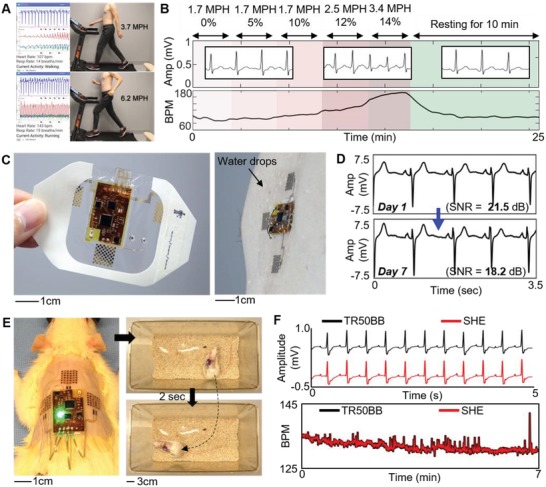
Demonstration of the SHE as a versatile clinical and research tool. A) Demonstration of the SHE application as ECG and HR monitor for a treadmill exercise including walking and running (Movie S6, Supporting Information). B) Implementation of a modified Bruce protocol (cardiac diagnostic test) and overlay of SHE's BPM data on plot areas whose color gradients represent the level of exercise intensity (top labels indicate speed and angle of inclination). C) Photos of a Tegaderm‐integrated SHE where the elastomer substrate has been substituted with a medical film (left), whose waterproof capability (right) enables a prolonged application exceeding 7 d with daily activities. D) Comparison of ECG collected at the beginning and end of the 7 d application of the device on the chest. No significant degradation in the signal qualities is observed with a decrease in SNR of only 3.3 dB. E) Demonstration of the SHE's utility in an in vivo animal study, involving ambulatory ECG monitoring of a rat model (left; device on the shaved back). With the ultrathin SHE on the back, this rat shows no change in activation patterns (right; after awakening from anesthesia; Movie S7, Supporting Information). A Tegaderm‐integrated SHE is applied for simultaneous recording of ECG with an implanted electronic system (TR50BB) of a rat with a high degree atrioventricular block. F) The ECG waveform and BPM data, generated by two devices, show nearly identical trends (Movie S8, Supporting Information).

As described in Figure 2, the SHE can be distinguished for its low‐profile integration with the skin without disrupting the wearer's behavior while preserving the quality of physiological signals despite movements of varying intensities. To fully depict its utility as a viable, versatile clinical and research tool, we employed a set of in vivo studies involving both humans and animals in varying contexts. First, we demonstrated the use of the SHE for real‐time monitoring of ECG and BPM during a treadmill test (**Figure** **5**A), which is known for its diagnostic and prognostic values in the subject's cardiac function. For this demonstration, we employed a modified Bruce protocol, which is consisted with five 3 min stages with progressively increasing workloads (determined by speed and incline) followed by 10 min of rest. Figure 5B captures the data recorded by the SHE throughout the 24 min protocol with top plot labels indicating the speed and inclination of each 3 min stage. The BPM data successfully describes the workload‐dependent changes in the heart rate with the peak effort recorded (BPM = 176) at the end of stage 5. While not directly part of a standard Bruce protocol, motion data could be successfully classified according to the subject's activities on a treadmill for “Idle,” “Walking,” and “Running” (details of the treadmill exercise appear in the Supporting Information, Figure S23 and Movie S6). The modular nature of the SHE allows the integration of alternative substrates other than elastomers depending on the application. Specifically, the utilization of a breathable medical tape, such as Tegaderm (3M), offers specific advantages of improved breathability and increased adhesion. Detail performance of the Tegaderm‐integrated device is shown in Figure S24 (Supporting Information). To demonstrate the advantages of the Tegaderm‐integrated SHE, we conducted a study verifying the feasibility of wearing the device for prolonged wear time up to 1 week (Figure S25), which is the suggested period for increasing the detection rates for subclinical arrhythmias and atrial fibrillation.^39^, ^40^ As shown in Figure 5C, the Tegaderm‐integrated SHE was applied to the chest of a subject, who was instructed to carry out daily tasks, such as walking, driving, showering, and sleeping until the noticeable degradation in the signals were observed. As shown in Figure 5D, the ECG waveforms between Day 1 and Day 7 showed no significant changes with a slight decrease in SNR by 3.3 dB due to the accumulated dead cells on epidermis.^28^ However, an abrupt decrease in SNR was observed in the 8^th^ day, where significant noise was visible in the waveform. This is likely due to eventual separation of the adhesive and electrodes from the skin. Photos describing the daily progression of the device's visual appearance and the waveforms describing the sudden changes in ECG quality are included in Figure S25 (Supporting Information). Ultimately, the Tegaderm‐integrated SHE presented the feasibility of continuous and prolonged ECG monitoring for 7 d with the advantages of the real‐time monitoring functionalities. Continuous cardiac monitoring of live animals is essential part in in vivo investigations involving various cardiac disease models. In such cases, the standard protocol is to surgically implant a biopotential telemeter in the animal along with lead wires for necessary ECG vectors. However, the invasive route is disadvantageous since the surgical procedure bears risks of the animal's health and life. Moreover, it requires a trained personnel and equipment necessary to perform the surgical procedure. Lastly, currently available invasive monitoring solutions are costly with telemeter units ranging ≈$3000 and require proprietary hardware for data acquisition, making the whole system cost of ≈$10 000. In this sense, the SHE‐based monitoring could contribute to studies involving live animals since it is non‐invasive, low‐cost, and does not obstruct the activity of the animals. However, the structure of an animal's skin is wildly different from the human's, which does not warrant the same conformal contact. Thus, the integration of the medical film, Tegaderm, is an attractive option since the adhesive can help secure the device regardless of the animal's skin conditions and the level of activities. To verify the feasibility of the SHE‐based in vivo cardiac monitoring, we applied the device over a shaved back of a high degree atrioventricular block model. Prior to the SHE application, a commercial, implantable telemeter unit (TR50BB, KAHA Sciences) was inserted in the abdominal cavity of the rat and sensing wire electrodes were sutured to the pectoral muscles (Figure 5E). As shown in Figure 5F, the two simultaneously recorded ECG data exhibited comparable performances. Similarly, the two BPM data, derived from the two devices, precisely overlapped one another, indicating the SHE's capability for advanced analysis involved with heart rhythms. Details of the experiment set up and data comparison between TR50BB and SHE are included in the Supporting Information (Figure S26 and Movies S7 and S8).

## 3 Discussion

For over a decade, the direct integration of electronics with soft biological tissues, such as human skin, with aims for persistent health monitoring has been a research topic for many. The most notable breakthrough was perhaps showcased by Kim et al. in 2011,^12^ in which the authors employed thin‐film processing techniques and stretchable interconnect schemes to match the mechanical properties of human skin and to serve as multifunctional healthcare platform. However, fundamental physics, data transmission, and reliability issues limit the formation of completely thin membrane electronics with performances that match commercially available rigid chip packages with active wireless telemetry. The hybrid electronic platform demonstrated by the SHE is an engineering solution to the technological gap that lies between “skin‐like properties” and “functionality.” The 500 µm thick, low modulus elastomer layer preserves the thin‐film properties of the nanomembrane Au electrodes and allows the stable monitoring of ECG without the use of a conductive gel. The mechanical decoupling chamber below the circuit's footprint helps separate the influence of the chip components on the electrodes and improves the overall adhesion of the SHE to skin. The decoupled structure therefore allowed us to inherit the merits of both thin, skin‐conforming electrodes and advanced circuit solutions, such as BLE, front‐end amplifier, and motion sensing. As shown throughout the manuscript, the thin and compact electronic component of SHE allowed us to design and optimize the adhesive property of the completed device according to each study. The extremely low modulus elastomer, such as Ecoflex1:2, is particularly attractive for its gentle adhesion and minimum impact on the skin. Consequently, we believe that the SHE will find its most utility in pediatric patients with congenital heart diseases, whose skin is too fragile and sensitive for repeated application and removal of adhesives and gels. Moreover, incorporation of a commercial medical adhesive as the substrate material highlighted the SHE's versatility in more demanding scenarios described in Figure 5, where the feasibility for wireless, prolonged, real‐time monitoring of cardiac abnormalities with aims for increased detection rates for early‐stage cardiac conditions is assessed. As a follow‐up validation study, the SHE's diagnostic efficiency should be assessed by comparing the detection rates with commercial devices in a clinical setting. The success of the comparison study will help both the patients and clinicians obtain the correct cardiac conditions and rule out false negatives and positives in outpatient settings. As described in Figure 5E,F, the successful monitoring of the rat model's cardiac activity points to future studies where the compact and low‐profile SHE enables a low‐cost, non‐invasive, and continuous monitoring of animal models. The real‐time ECG classification algorithm could be designed to detect not only the abnormalities, but also the onset of action for the administered drugs. Such smart functionality could provide the researchers an objective tool and the better control over animal experiments. In this paper, MLV2 was used as an analog to precordial lead V2 using two bipolar surface electrodes. In order to demonstrate the precision of real‐time annotation, we demonstrated the use of convolutional neural networks trained on two publicly available 12‐lead ECG datasets, using only the V2 lead. We trained a separate model recording ECG using MLV2 demonstrating similar ability to detect arrhythmia and annotate ectopic beats in real‐time. We demonstrated this capability with a subject exhibiting a symptom of VEB (specifically, a premature ventricular contraction). However, there is not enough data recorded with our MLV2 lead to create a fully fledged annotation model, as the one used for the V2 leads from the PTB and INCART datasets, covering nine classes. In future studies, we aim to acquire ECG data from nine or more classes using MLV2 with the SHE to ensure high‐quality recordings in at‐home idle and ambulatory settings. This will allow for a more robust annotation and classification scheme that can be used in any at‐home setting with reliable detection and accuracy.

## 4 Conclusion

The collection of materials presented here indicates that the wireless SHE offers real‐time, smart, and ambulatory monitoring of human health. This work includes a comprehensive study that lays the technological basis for settings where persistent and multifunctional health monitoring is desirable with minimal impact on the patient's condition and lifestyle. The persistent, yet comfortable, integration of SHE‐type monitors, along with the continuous and wireless acquisition of multiple health conditions, will serve as a powerful assistance to the practice of medicine.

## 5 Experimental Section

*Elastomer Substrate Preparation*: Polystyrene dishes (FB0875714, Fisher Scientific) were used as cast molds for elastomer preparation. A freshly mixed elastomer (9.8 g) was poured onto the dish to achieve the 500 µm thickness. For elastomers with short pot life, such as Ecoflex1:1 and 1:2, a spin coater was used to help distribute the material evenly across the dish. Cured elastomers were cut and removed from the dish for thin‐film integration.

*Microfabrication of Thin‐Film Structures and Transfer onto Elastomer*: Standard microfabrication processes, such as photolithography, wet/dry etching, physical vapor deposition, spin coating, and vacuum oven curing, were used to construct the thin‐film metal/polymer composite layers (electrodes, interconnection, and connector). Details of the fabrication steps appear in Supporting Information Note 1. Completed thin films were peeled from the donor substrates (PDMS‐coated silicon wafers) using a water‐soluble tape (ASWT‐2, Aquasol). The backsides of the thin films were deposited with layers of Ti (5 nm) and SiO_2_ (20 nm) using an electron beam deposition tool. Elastomer substrates were treated with a UV‐ozone dry stripper (UV‐1, Samco) for 5 s for surface activation. The thin films were then laminated to the activated elastomer and gently pressed to form the —O—Si—O— covalent bonds. Water soluble tape was removed by rinsing with water and foam tip swabs, leaving behind the bonded thin‐film structure on the elastomer.

*SNR Calculation for ECG*: SNR measurements were made by splitting an ECG recording into windows of 8 s in length, where the peaks were isolated using a variation on the Pan‐Tompkins algorithm for detecting the QRS complex.^41^ After the convolutional step (which can be seen in Figure 3B, first column), where the relative amplitude of the signal peaks is found, the peaks were removed using a high‐order median filter (order = 99), leaving only the relevant noise. The signal‐to‐noise ratio was then calculated using the average amplitude of each QRS complex versus the average noise amplitude. The SNR of a single window was calculated as follows ${\text{SNR}}_{\text{dB}}\;\;=\;\;10\;\log_{10}\left[{\left(\frac{A_{\text{signal}}}{A_{\text{noise}}}\right)}^{2}\right]$, and was averaged over the number of windows in the recording.

*In Vivo Experiment with Human Subjects*: The study involved eight volunteers ages 18–40 and the study was conducted by following the approved IRB protocol (# H17212) at Georgia Institute of Technology. Prior to the in vivo study, all subjects agreed with the study procedures and provided signed consent forms.

*In Vivo Experiment with Animals*: The study involved two rats and the study was conducted by following the approved IACUC protocol #2003043 “Cardiac rhythm restoration in a small animal model of heart disease” at Emory University.

## Conflict of Interest

Georgia Tech has a pending US patent application regarding the stretchable hybrid electronics.

## Supporting information

Supplementary

Supplementary

Supplementary

Supplementary

Supplementary

Supplementary

Supplementary

Supplementary

Supplementary
