# Supplementary material for: All‐in‐One, Wireless, Stretchable Hybrid Electronics for Smart, Connected, and Ambulatory Physiological Monitoring
Source: Adv Sci (Weinh). 2019 Jul 24;6(17):1900939. doi: 10.1002/advs.201900939 (PMC6724359; doi:10.1002/advs.201900939)

Copyright WILEY-VCH Verlag GmbH & Co. KGaA, 69469 Weinheim, Germany, 2019.

ADVANCED SCIENCE
Open Access

Supporting Information

for *Adv. Sci.,* DOI: 10.1002/advs.201900939

All-in-One, Wireless, Stretchable Hybrid Electronics for Smart, Connected, and Ambulatory Physiological Monitoring

Yun-Soung Kim, Musa Mahmood, Yongkuk Lee, Nam Kyun Kim, Shinjae Kwon, Robert Herbert, Donghyun Kim, Hee Cheol Cho, and Woon-Hong Yeo**

Copyright WILEY-VCH Verlag GmbH & Co. KGaA, 69469 Weinheim, Germany, 2018.

Supporting Information

All-in-One, Wireless, Stretchable Hybrid Electronics for Smart, Connected, and Ambulatory Physiological Monitoring

Yun-Soung Kim, Musa Mahmood, Yongkuk Lee, Nam Kyun Kim, Shinjae Kwon, Robert Herbert, Donghyun Kim, Hee Cheol Cho, and Woon-Hong Yeo**

Dr. Y.-S. Kim, M. Mahmood, S. Kwon, R. Herbert, Prof. D. Kim, Prof. W.-H. Yeo
George W. Woodruff School of Mechanical Engineering, Institute for Electronics and Nanotechnology, Georgia Institute of Technology, Atlanta, GA 30332, USA

Prof. Y. Lee
Department of Biomedical Engineering, Wichita State University, Wichita, KS 67260, USA

Dr. N. K. Kim
Department of Pediatrics, School of Medicine, Emory University, Atlanta, GA 30322, USA
Department of Pediatrics, Yonsei University College of Medicine, Seoul 03722, South Korea

Prof. D. Kim
Department of Surgery, Yonsei University Wonju College of Medicine, Wonju, Gangwon-do 220701, South Korea

Prof. H. C. Cho, Prof. W.-H. Yeo
Wallace H. Coulter Department of Biomedical Engineering, Parker H. Petit Institute for Bioengineering and Biosciences, Georgia Institute of Technology and Emory University, Atlanta, GA 30332, USA
E-mail: heecheol.cho@emory.edu (H. C. Cho)

Prof. W.-H. Yeo
Center for Flexible and Wearable Electronics Advanced Research, Institute for Materials, Neural Engineering Center, Georgia Institute of Technology, Atlanta, GA 30332, USA
E-mail: whyeo@gatech.edu (W.-H. Yeo)

Keywords: wearable electronics, stretchable hybrid electronics, ambulatory cardiac monitoring, physiological signals

Note S1. Microfabrication of thin-film metal/polymer composite structures

a. Electrode fabrication

1. Spincoat PDMS at 3000 rpm for 30 sec.
2. Spincoat 1st polyimide (PI, PI-2610, HD MicroSystems) at 5000 rpm for 1 min.
3. Cure PI in a vacuum oven at 300 °C for 1 hr.
4. Deposit 5 nm-thick Cr and 100 nm-thick Au by sputtering.
5. Spincoat photoresist (PR, Microposit SC1827, MicroChem) at 3000 rpm for 30 sec.
6. Bake PR on a hot plate at 100 °C for 2 min.
7. Align with a photomask and expose UV light, intensity of 15 mJ/cm2, for 7 sec.
8. Pattern metal layers with electrode design using photolithography.
9. Develop exposed PR with a developer (AZ 300MIF, Integrated Micro Materials).
10. Etch exposed Cr with Cr etchant (Chrome Mask Etchant 9030, Transene).
11. Etch exposed Au with Au etchant (GE-8110, Transene).
12. Etch exposed PI using reactive ion etcher (RIE) at 150 W, 150 mTorr, and 20 sccm of O_2 for 13 min.
13. Remove residual materials with acetone and foam-tip swabs.
14. Rinse with IPA and deionized DI water.

b. Flexible interconnection circuit fabrication

1. Spincoat PDMS at 3000 rpm for 30 sec.
2. Spincoat 1st PI (PI-2610, HD MicroSystems) at 5000 rpm for 1 min.
3. Cure PI in a vacuum oven at 300 °C for 1 hr.
4. Deposit 500 nm-thick Cu by sputtering.
5. Spincoat PR (Microposit SC1827, MicroChem) at 3000 rpm for 30 sec.
6. Bake PR on a hot plate at 100 °C for 2 min.
7. Align with a photomask and expose UV light, intensity of 15 mJ/cm2, for 7 sec.
8. Develop exposed PR with a developer (AZ 300MIF, Integrated Micro Materials).
9. Etch exposed Cu with Cu etchant (APS-100, Transene) diluted with DI water (APS-100:DI water=2:1).
10. Strip PR with acetone and foam swabs, followed by IPA and DI water rinse.
11. Dehydration on a 100 °C hot plate for 1 min.
12. Spincoat the first layer of 2nd PI (PI-2524, HD MicroSystems) at 950 rpm for 1 min.
13. Cure PI on a hot plate at 100 °C for 5 min and in a vacuum oven at 250 °C for 3 hr including ramping from room temperature.

14. Spincoat the second layer of 2nd PI at 950 rpm for 1 min.
15. Cure PI on a hot plate at 100 °C for 5 min and in a vacuum oven at 250 °C for 3 hr including ramping from room temperature.
16. Spincoat PR (AZ P4620, Integrated Micro Materials) at 1000 rpm for 30 sec.
17. Bake PR on a hot plate at 90 °C for 4 min.
18. Align with a photomask and expose UV light, intensity of 15 mJ/cm2, for 2 min.
19. Develop exposed PR with a developer (AZ 400K, Integrated Micro Materials) diluted with DI water (AZ 400K:DI water=1:3).
20. Etch exposed PI using reactive ion etcher (RIE) at 150 W, 150 mTorr, and 20 sccm of O2 for 45 min.
21. Strip PR with acetone and foam swabs, followed by IPA and DI water rinse.
22. Dehydration on a 100 °C hot plate for 1 min.
23. Deposit 1.5 µm-thick Cu by sputtering.
24. Spincoat PR (AZ P4620) at 2000 rpm for 30 sec.
25. Bake PR on a hot plate at 90 °C for 4 min.
26. Align with a photomask and expose UV light, intensity of 15 mJ/cm2, for 80 sec.
27. Develop exposed PR with a developer (AZ 400K) diluted with DI water (AZ 400K:DI water=1:3).
28. Etch exposed Cu with Cu etchant (APS-100) diluted with DI water (APS-100:DI water=2:1).
29. Strip PR with acetone and foam swabs, followed by IPA and DI water rinse.
30. Dehydration on a 100 °C hot plate for 1 min.
31. Spincoat 3rd PI (PI-2524) at 2000 rpm for 1 min.
32. Cure PI on a hot plate at 100 °C for 5 min and in a vacuum oven at 250 °C for 3 hr including ramping from room temperature.
33. Spincoat PR (AZ P4620) at 2000 rpm for 30 sec.
34. Bake PR on a hot plate at 90 °C for 4 min.
35. Align with a photomask and expose UV light, intensity of 15 mJ/cm2, for 80 sec.
36. Develop exposed PR with a developer (AZ 400K) diluted with DI water (AZ 400K:DI water=1:3).
37. Etch exposed PI using RIE at 150 W, 150 mTorr, and 20 sccm of O_2 for 18 min.
38. Strip PR with acetone and foam swabs, followed by IPA and DI water rinse.

c. Stretchable connector fabrication

1. Spincoat PDMS at 3000 rpm for 30 sec.
2. Spincoat 1st PI (PI-2610, HD MicroSystems) at 5000 rpm for 1 min.

3. Cure PI in a vacuum oven at 300 °C for 1 hr.
4. Deposit 500 nm-thick Cu by sputtering.
5. Spincoat PR (Microposit SC1827) at 3000 rpm for 30 sec.
6. Bake PR on a hot plate at 100 °C for 2 min.
7. Align with a photomask and expose UV light, intensity of 15 mJ/cm2, for 7 sec.
8. Develop exposed PR with a developer (AZ 300MIF).
9. Etch exposed Cu with Cu etchant (APS-100, Transene) diluted with DI water (APS-100:DI water=2:1).
10. Strip PR with acetone and foam swabs, followed by IPA and DI water rinse.
11. Dehydration on a 100 °C hot plate for 1 min.
12. Spincoat 2nd PI (PI-2524) at 5000 rpm for 1 min.
13. Cure PI on a hot plate at 100 °C for 5 min and in a vacuum oven at 250 °C for 3 hr including ramping from room temperature.
14. Deposit 500 nm-thick Cu by sputtering.
15. Spincoat PR (Microposit SC1827) at 3000 rpm for 30 sec.
16. Bake PR on a hot plate at 100 °C for 2 min.
17. Align with a photomask and expose UV light, intensity of 15 mJ/cm2, for 7 sec.
18. Develop exposed PR with a developer (AZ 300MIF).
19. Etch exposed Cu with Cu etchant (APS-100) diluted with DI water (APS-100:DI water=2:1).
20. Strip PR with acetone and foam swabs, followed by IPA and DI water rinse.
21. Dehydration on a 100 °C hot plate for 1 min.
22. Spincoat 3rd PI (PI-2524) at 5000 rpm for 1 min.
23. Cure PI on a hot plate at 100 °C for 5 min and in a vacuum oven at 250 °C for 3 hr including ramping from room temperature.
24. Spincoat PR (AZ P4620) at 2000 rpm for 30 sec.
25. Bake PR on a hot plate at 90 °C for 4 min.
26. Align with a photomask and expose UV light, intensity of 15 mJ/cm2, for 80 sec.
27. Develop exposed PR with a developer (AZ 400K) diluted with DI water (AZ 400K:DI water=1:3).
28. Etch exposed PI using RIE at 150 W, 150 mTorr, and 20 sccm of O_2 for 20 min.
29. Strip PR with acetone and foam swabs, followed by IPA and DI water rinse.

Note S2. Derivation of the conformal contact criteria for 500 µm-thick elastomer substrates

To determine elastomer substrates that provide support and conformal contact, the interface mechanics between the electrode and skin were analytically modeled. We assume a sinusoidal skin surface described as:

$$y(x) = \frac{h_{rough}}{2}\left(1+\cos\frac{2\pi x}{\lambda_{rough}}\right) \quad (1)$$

Here, y(x) is skin roughness, h_{rough} is roughness amplitude, and λ_{rough} is wavelength. During non-conformal contact, the total energy at the interface is approximately zero due to lack of contact area. Conformal contact results in the electrode and skin surface aligning exactly. Thus, electrode displacement ($w(x)$) and skin displacement ($u_z(x)$) is given as:

$$w(x) = \frac{h}{2}\left(1+\cos\frac{2\pi x}{\lambda_{rough}}\right) \quad (2)$$

$$u_z(x) = y - w = \frac{h_{rough}-h}{2}\left(1+\cos\frac{2\pi x}{\lambda_{rough}}\right) \quad (3)$$

The maximum deflection of the electrode is h .

Conformal contact conditions are determined by calculating interfacial contact energy ($U_{conformal}$) by:

$$U_{conformal} = U_{bending} + U_{skin} + U_{adhesion} \quad (4)$$

Electrode bending energy, skin elastic energy, and contact adhesion energy are represented by $U_{bending}$, U_{skin} , and $U_{adhesion}$, respectively.

Bending energy is calculated as:

$$U_{bending} = \frac{1}{\lambda_{rough}}\int_0^{\lambda_{rough}} \frac{EI(w'')^2}{2}dx = \frac{\pi^4 EIh^2}{\lambda_{rough}^4} \quad (5)$$

Here, the electrodes and substrate are modeled as a composite material where the effective bending stiffness (EI) is determined as:

$$EI = \alpha EI_{electrode} + (1-\alpha)EI_{elastomer} \quad (6)$$

$$EI_{electrode} = \sum_{i=1}^{N} E_i h_i \left[\left(b - \sum_{j=1}^{i} h_j\right)^2 + \left(b - \sum_{j=1}^{i} h_j\right)h_i + \frac{1}{3}h_i^2\right] \quad (7)$$

$$EI_{elastomer} = \frac{E_{elastomer} h_{elastomer}^3}{12} \quad (8)$$

where

$$b = \sum_{i=1}^{N} E_i h_i \left(\sum_{j=1}^{i} h_j - \frac{1}{2}h_i\right) / \sum_{i=1}^{N} E_i h_i \quad (9)$$

The areal fraction of PI and Au is α . $E_{elastomer}$ and $h_{elastomer}$ is the Young's modulus and thickness, respectively, of the elastomer substrate. The bending stiffness of the electrode pattern ($EI_{electrode}$) is calculated as a composite of N layers, and E_i is the Young's modulus of the ith layer and h_i is the thickness of the ith layer.

The elastic energy of skin is determined as:

$$U_{skin} = \frac{1}{\lambda_{rough}}\int_0^{\lambda_{rough}} \frac{\sigma_z u_z}{2} dx = \frac{\pi E_{skin}(h_{rough} - h)^2}{16\lambda_{rough}} \quad (10)$$

Where the normal stress of the skin surface is:

$$\sigma_z = \frac{\pi E_{skin}(h_{rough} - h)}{2\lambda_{rough}} \cos\frac{2\pi x}{\lambda_{rough}} \quad (11)$$

Adhesion energy is calculated as:

$$U_{adhesion} = -\gamma\int_0^{\lambda_{rough}} \sqrt{1+(w')^2}\, dx \approx -\gamma\left(1+\frac{\pi^2 h^2}{4\lambda_{rough}^2}\right) \quad (12)$$

for the case $\lambda_{rough} = 7h_{rough}$. The total work of adhesion is calculated as:

$$\gamma = \left(1-\alpha\right)\gamma_{elastomer} \quad (13)$$

where $\gamma_{elastomer}$ is the work of adhesion of the elastomer.

Conformal contact occurs when the adhesion energy is larger than the combination of bending and elastic energy. Substituting into the energy equation and solving for $\gamma_{elastomer}$ yields:

$$\gamma_{elastomer} > \left(\frac{1}{1-\alpha}\right)\frac{\frac{\pi^4 EIh^2}{\lambda_{rough}^4} + \frac{\pi E_{skin}\left(h_{rough}-h\right)^2}{16\lambda_{rough}}}{\left(1+\frac{\pi^2 h^2}{4\lambda_{rough}^2}\right)} \quad (14)$$

In the present study, skin conditions are h_{rough} = 55 µm, λ_{rough} = 385 µm, and E_{skin} = 130 kPa. The maximum α of the electrode is 0.543.

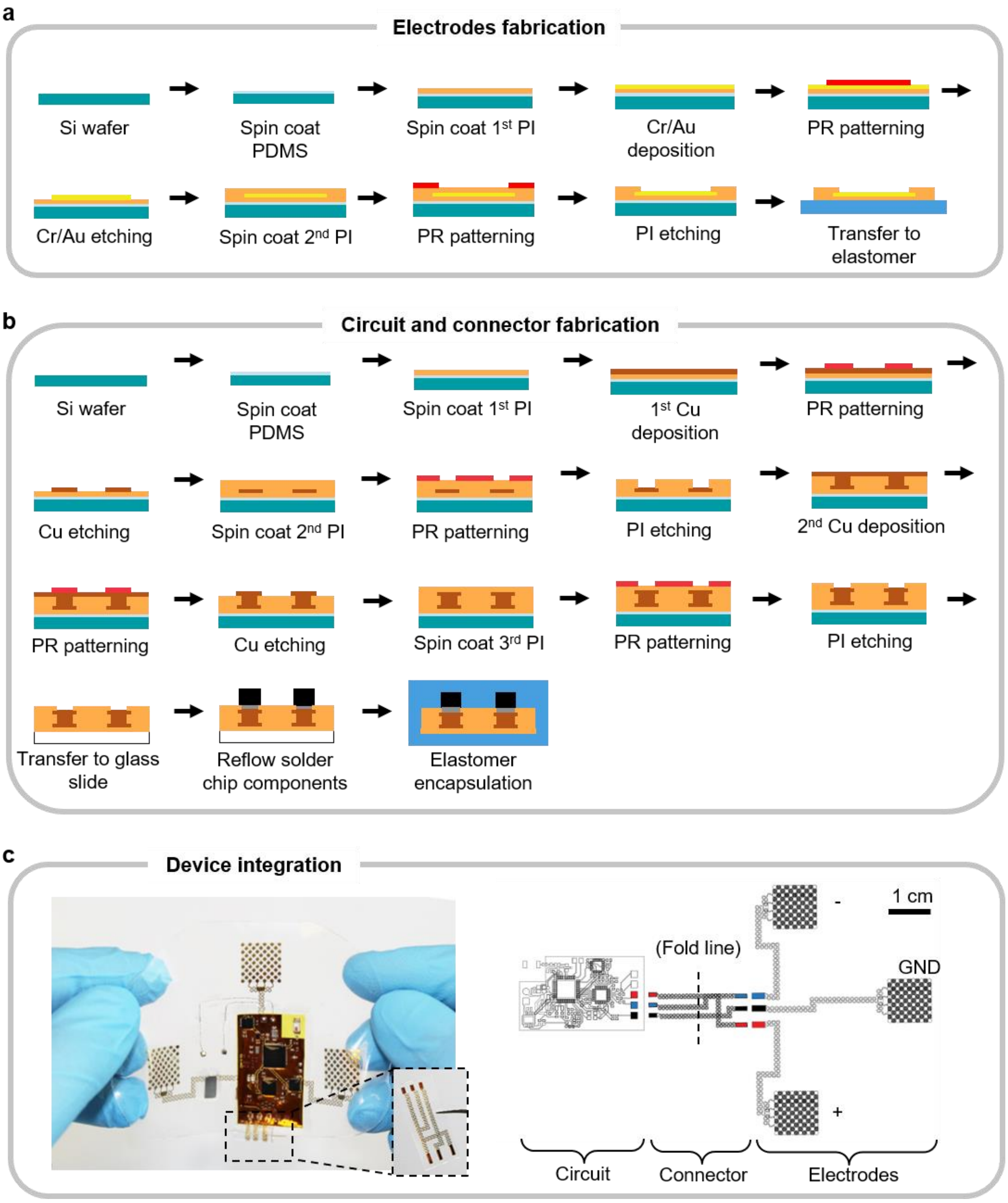

Figure S1. Assembly of SHE. (a) Fabrication process of the stretchable thin-film Au electrodes. (b) Fabrication process of the thin-film flexible circuit and stretchable connector. (c) (Left) Photo of a completed SHE. The zoomed inset shows the location of the stretchable connector. (Right) Diagram describing the connectivity of the electrodes to the input pads of the circuit.

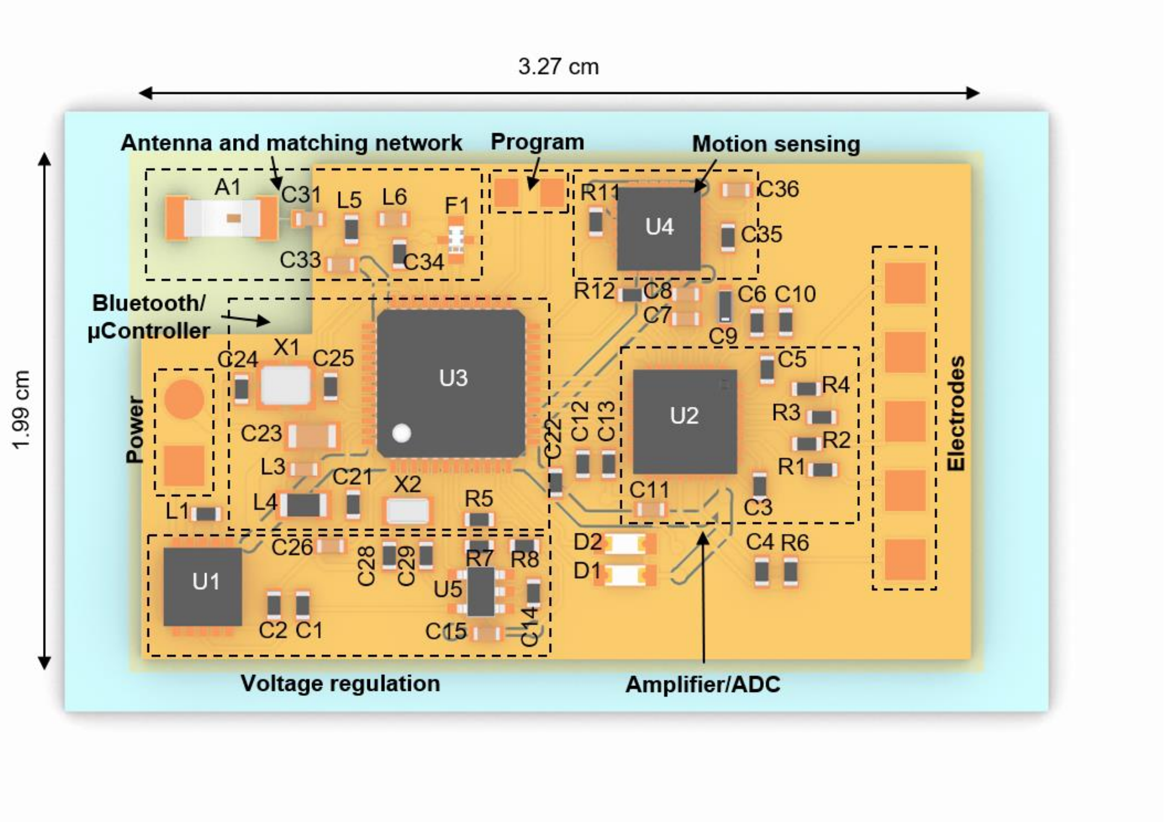

Figure S2. Circuit design. Top-view illustration of SHE's circuit component with highlighted functional blocks. Detail list of the surface mount chip components can be found in Table S1.

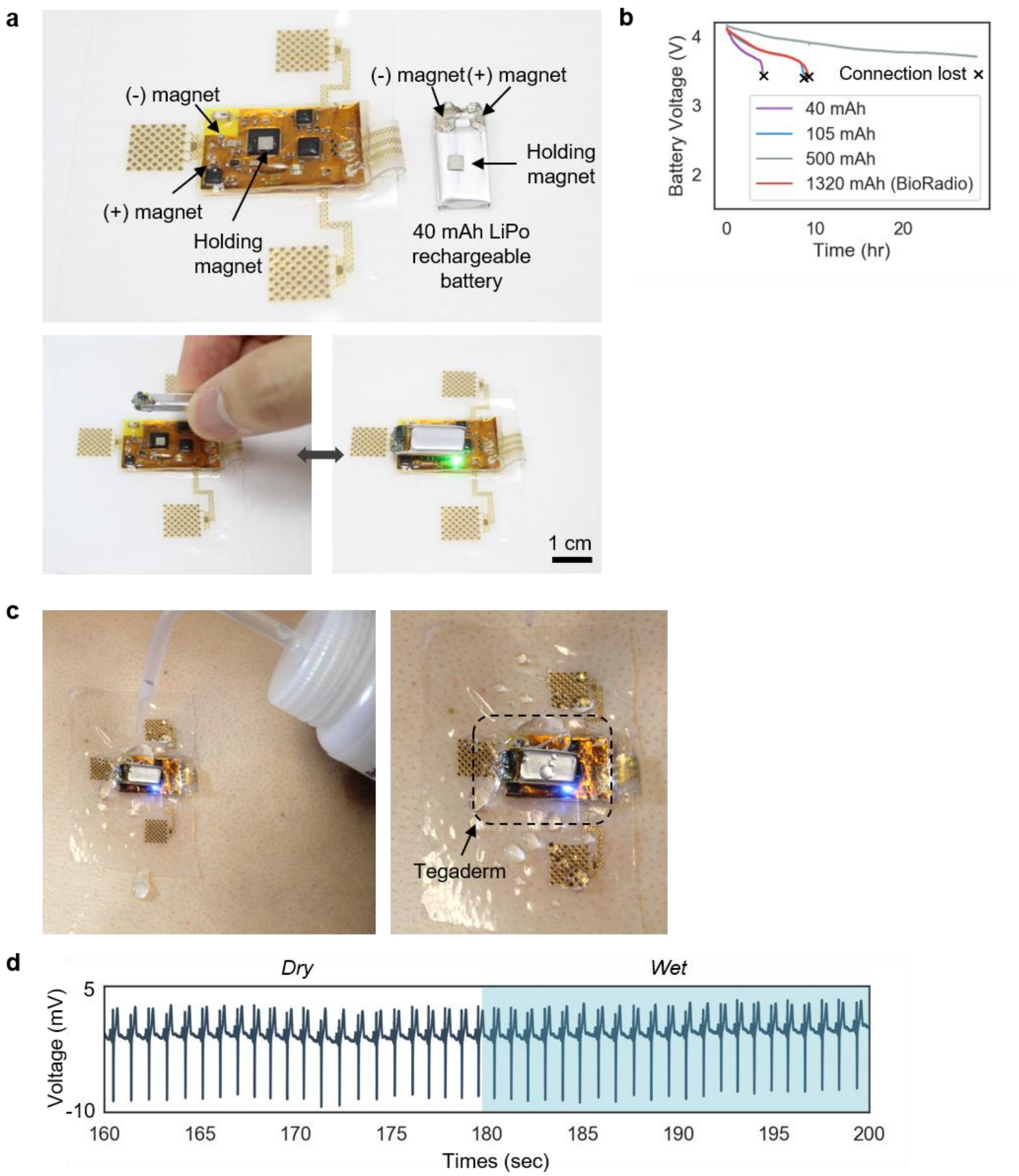

Figure S3. Magnetic integration of the battery and waterproofing strategy. (a) Photographs detail the placement for the neodymium magnets for holding and connecting the battery. (b) Battery life tests with various capacities. Average efficiency of the SHE is 0.1 hr/mAh. The data for 500 mAh battery is cut off at 30 hours due to the limitation in the recording system. (c) A layer of Tegaderm can be applied over the circuit for continuous acquisition of physiological data during showering. (d) The ECG measured before and after the application of water.

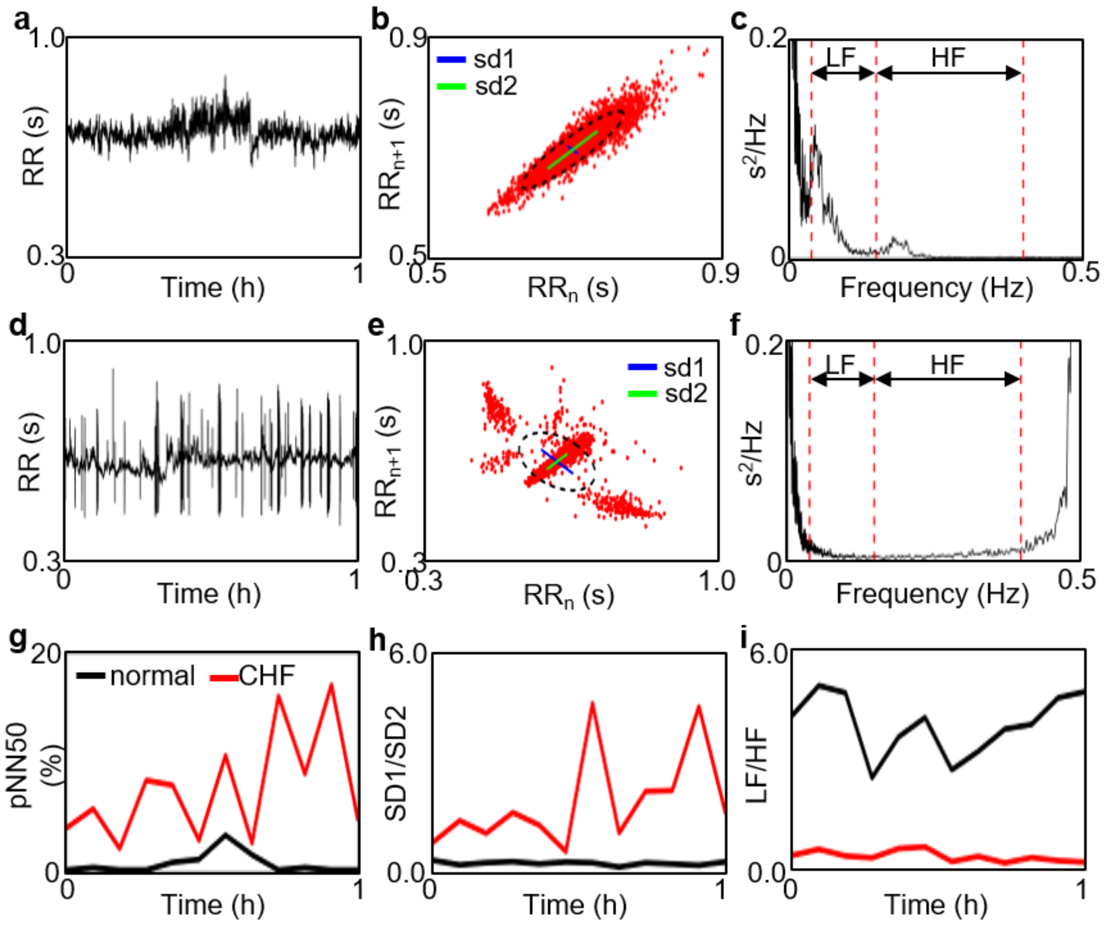

Figure S4. ECG analysis with clinical relevance. ECG acquired by SHE from a healthy subject is analyzed for (a) RR vs. time, (b) RR_{n+1} vs. RR_n, and (c) frequency spectrum. ECG heart failure patients obtained from an online database (PhysioNet) is analyzed for (d) RR vs. time, (e) RR_{n+1} vs. RR_n, and (f) frequency spectrum. Comparison between the critical heart failure (CHF) data and normal (SHE) data in (g) pNN50, (h) SD1/SD2, and (i) LF/HF analyses. (NN intervals: intervals between normal R peaks, but in practice, RR intervals and NN intervals are synonymous; pNN50: percentage of differences between adjacent NN intervals that are greater than 50 ms; SD1 and SD2: standard deviations of points perpendicular to the axis; Poincaré plot: a graph in which each RR interval is plotted against next RR interval. Research articles reported the potential of the plot in visual detection of heart-related diseases or abnormalities; LF/HF: ratio of low to high frequency power of all NN intervals. For more complete information, refer to https://physionet.org/tutorials/hrv-toolkit/ and https://physionet.org/physiotools/mpp/.)

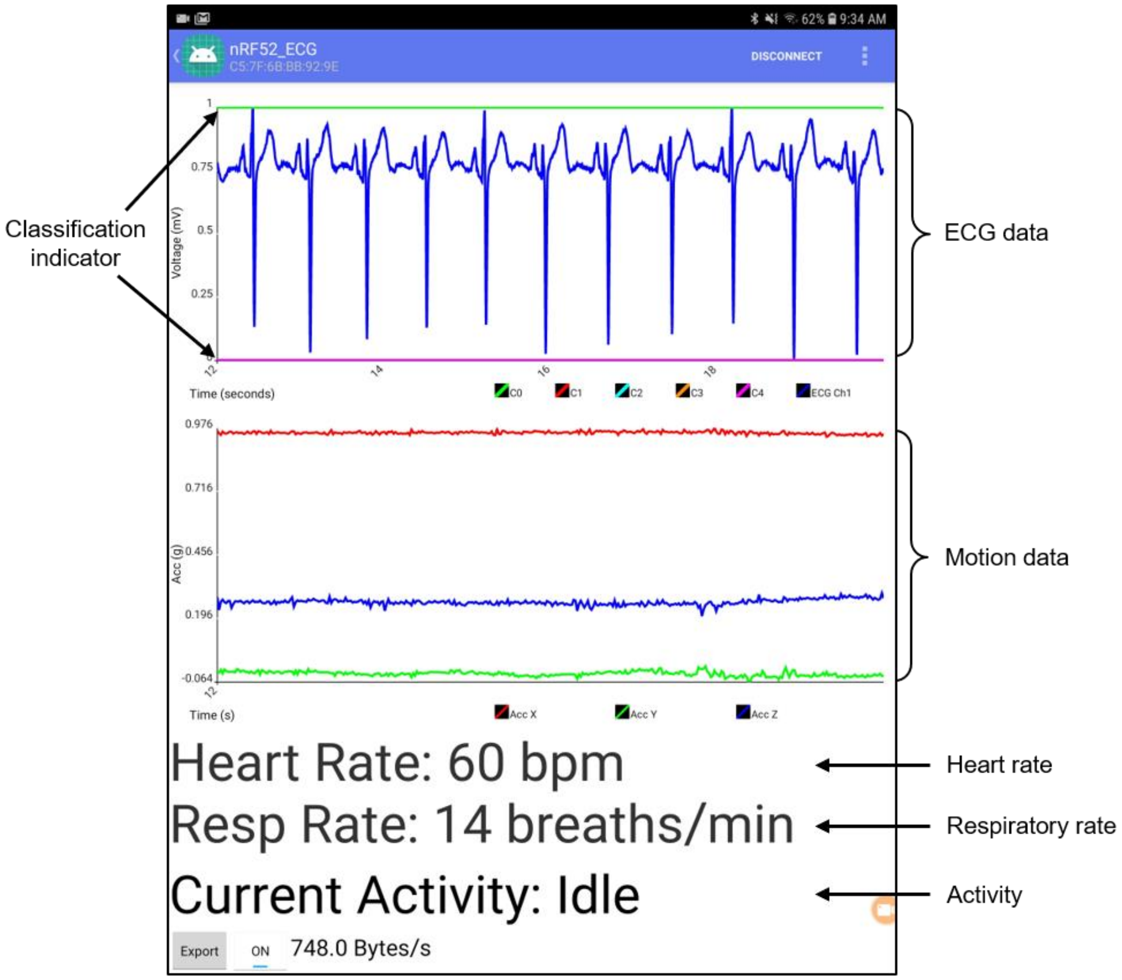

Figure S5. Application user interface. The screenshot captures the layout of the Android application. Raw ECG is display at the top and refreshed every 4 seconds. The real-time ECG classification result is displayed as an overlay colored line. Raw acceleration data is displayed in the middle of the screen and refreshed every second. Bottom texts include the real-time display of heart/respiratory rates and activity.

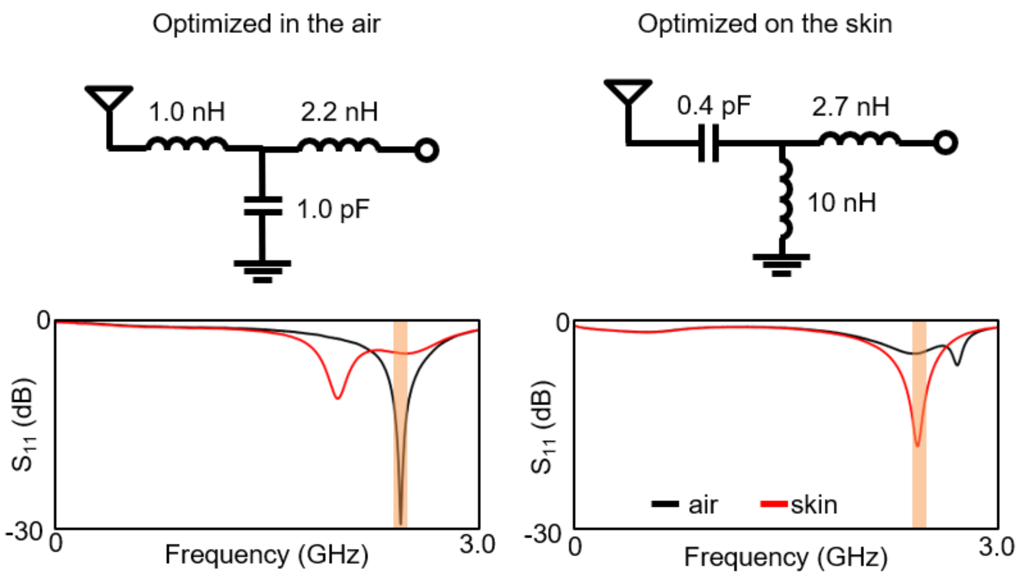

Figure S6. Antenna matching for on-skin application. The SHE's antenna property was optimized to maintain long-distance connectivity by characterizing the circuit's RF properties while positioned on the chest. T-matching network with series connections of 0.4 pF capacitor and 2.7 nH inductor and a parallel connection of a 10 nH inductor was able to shift the resonant frequency of SHE to 2.45 GHz while positioned on the chest.

a b

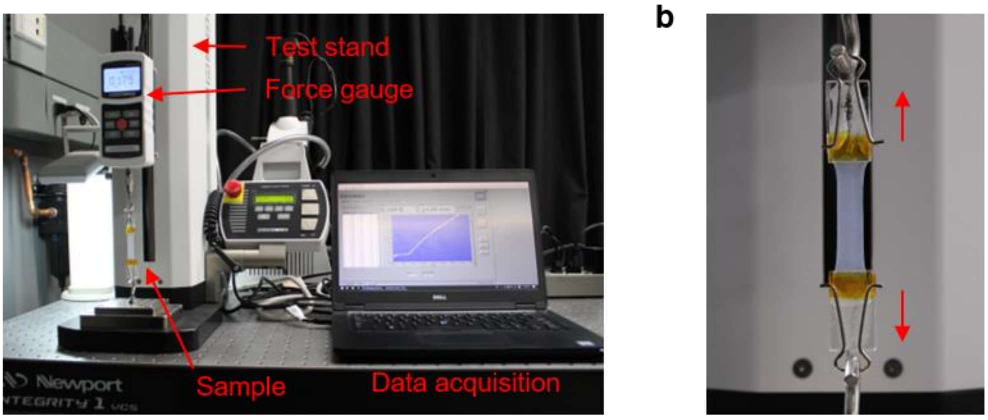

c

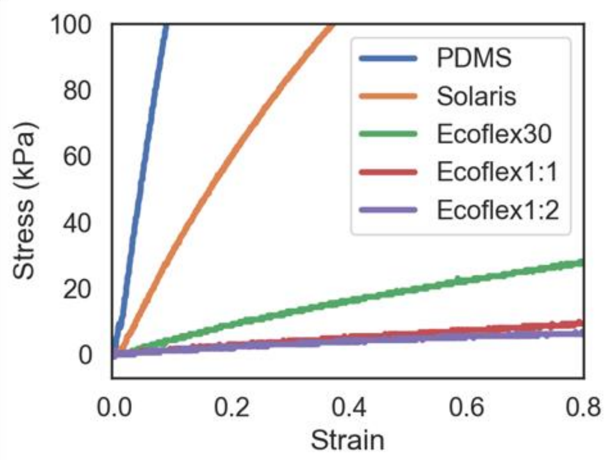

d

Elastomer	Young's modulus (kPa)
PDMS	1131.04
Solaris	251.53
Ecoflex30	32.23
Ecoflex1:1	11.17
Ecoflex1:2	7.85

Figure S7. Measurement of elastomer tensile modulus. (a) Measurement set up for elastomer tensile modulus. (b) Close-up view of a sample specimen. (c) Stress vs. strain curves of the 5 elastomer samples. (d) Young's moduli determined by finding the slope of each curve in its linear regime.

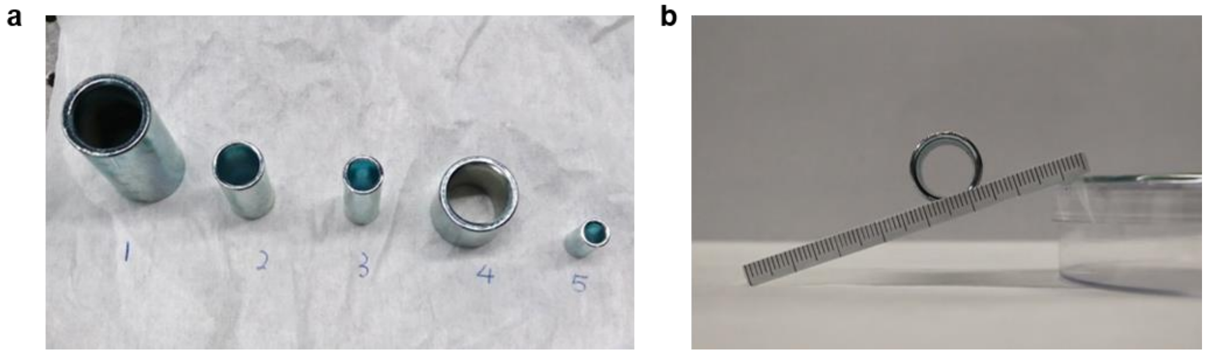

Figure S8. Measurement of elastomer work of adhesion. (a) 5 steel cylinders with different dimensions and weights were used. Summary of $\Delta\gamma$ values based on the cylinder's length, weight, and angles of the incline can be found in Table S2. (b) A snapshot of a cylinder rolling down an elastomer coated track.

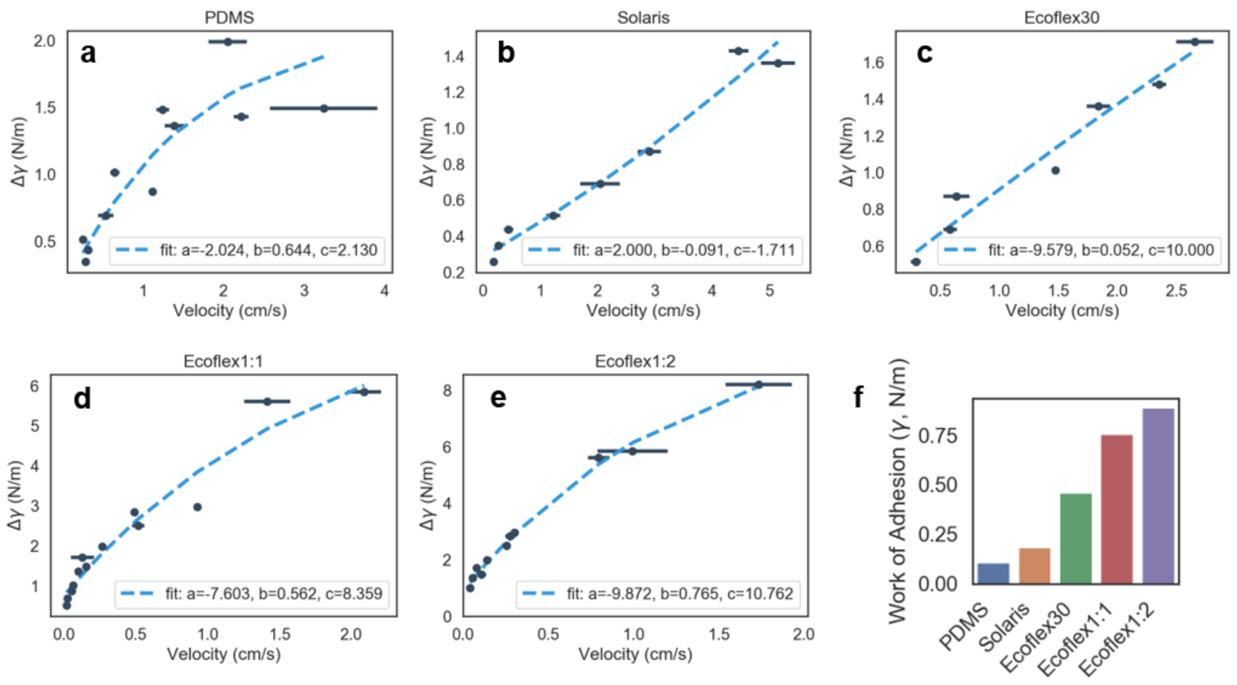

Figure S9. Determination of work of adhesion. (a-e) Curve fit lines and their y-intercept reveals the work of adhesion value for each elastomer. (f) Bar plot summarizing work of adhesion values of the five elastomers.

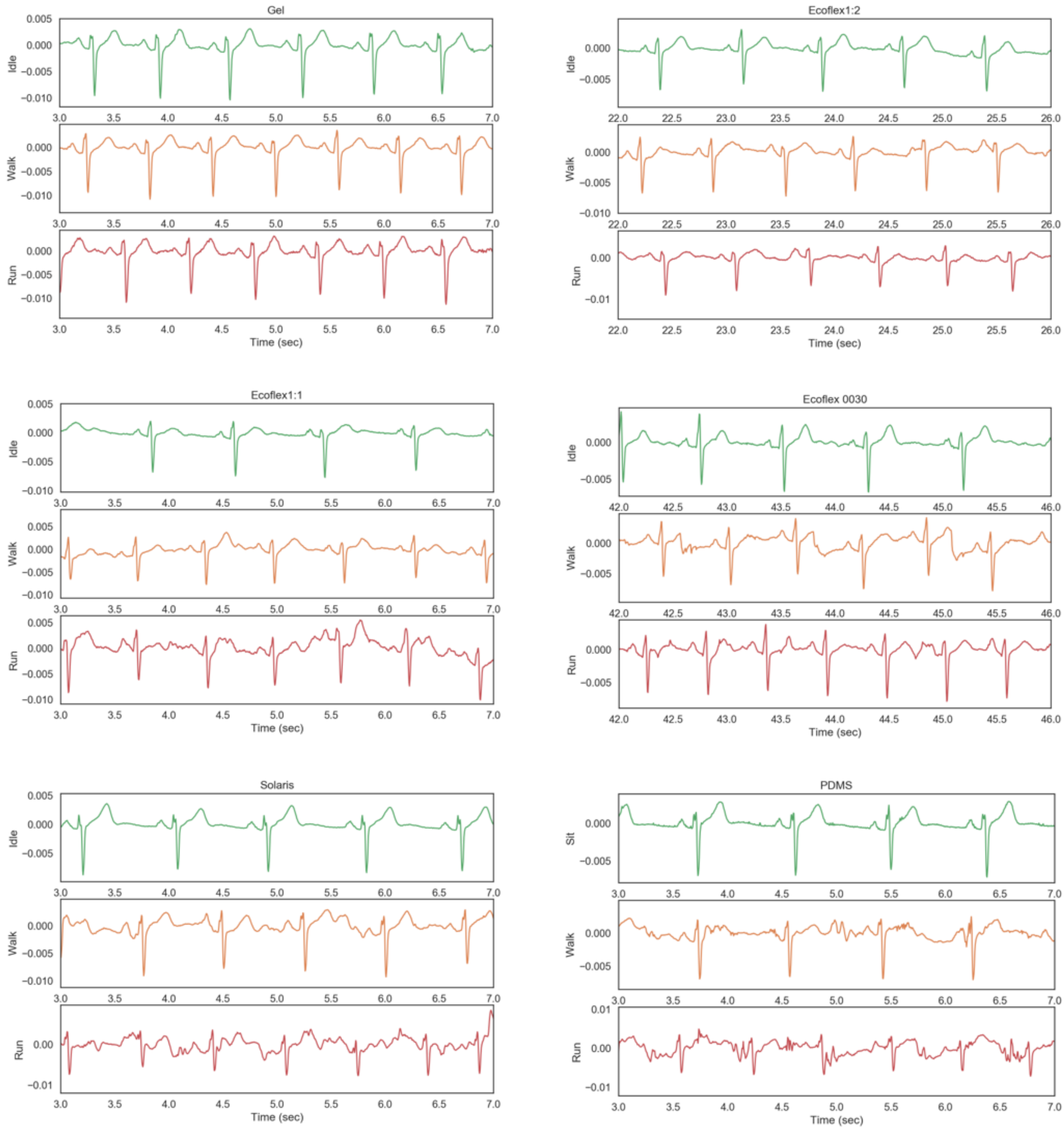

Figure S10. ECG waveforms of various electrodes. Four-second snapshots of all ECG waveforms collected from the thin-film electrodes integrated on five different elastomer substrates and a set of hydrogel electrodes. Interestingly, the Ag/AgCl hydrogel electrodes (top left data, MVAP-II Electrodes, MVAP Medical Supplies), which we used as a commercial reference showed superior impedance of 3.9 kΩ but showed slightly lower SNR (19.50 dB, 18.07 dB, 16.99 dB for sit, walk, run, respectively) compared to Ecoflex1:2. This is possibly due to the effectively larger contact area of the gel electrode contributing to improved impedance, but also to its less conformal property and vibration of the embedded metal tab lowering the SNR.

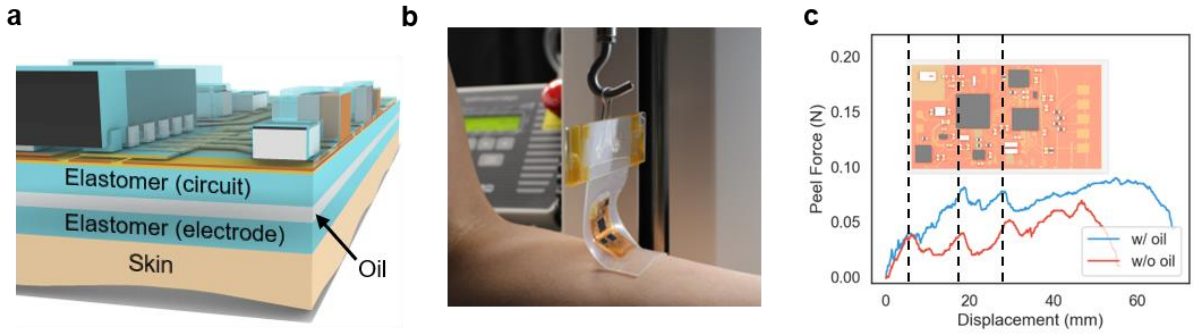

Figure S11. Effect of decoupling chamber. (a) Cross-section rendering shows the layer of oil-filled decoupling chamber between the two elastomer layers (between electrode and circuit sides). (b) Photo capturing the peel force measurement on a forearm. (c) Comparison of peel force plots between SHE specimen with and without a decoupling chamber.

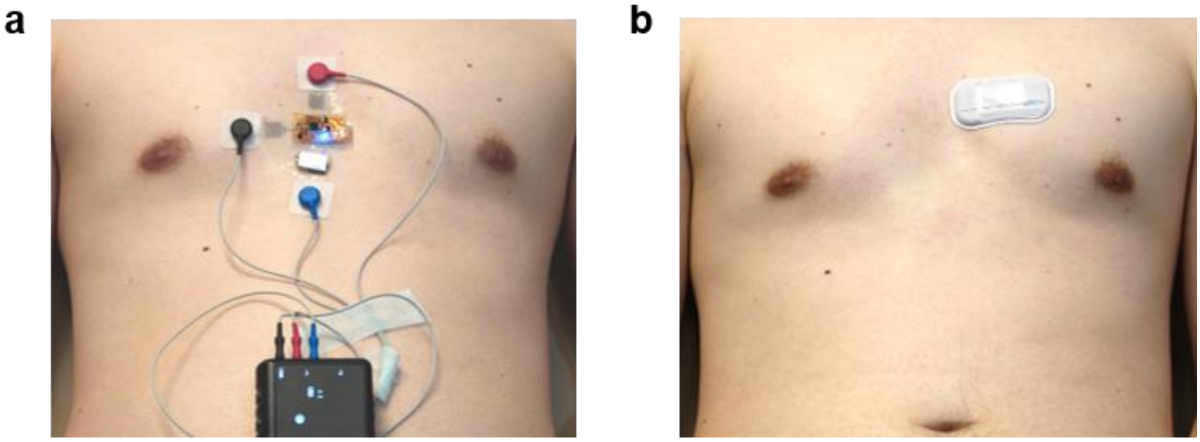

Figure S12. Comparison of ECG qualities. (a) SHE and BioRadio electrodes can be positioned on the chest simultaneously for a concurrent recording. (b) BioStamp was used separately due to the overlap in the application site.

a

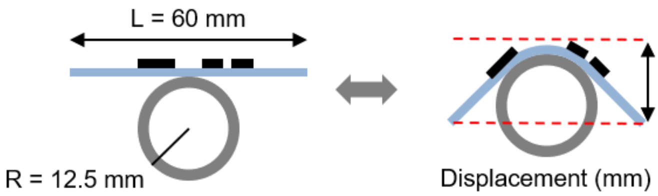

b

Trial	Cycle #	Displacement (mm)
1	1000	5.08
2	1000	7.62
3	1000	10.16
4	1000	12.70
5	1000	15.24

Figure S13. Cyclic stress test of SHE. (a) As shown in the schematic the portion of SHE containing the electronics was cyclically stressed by displacing it vertically using a cylinder. (b) Summary of the cyclic stress test protocol with the progressively increasing vertical displacement.

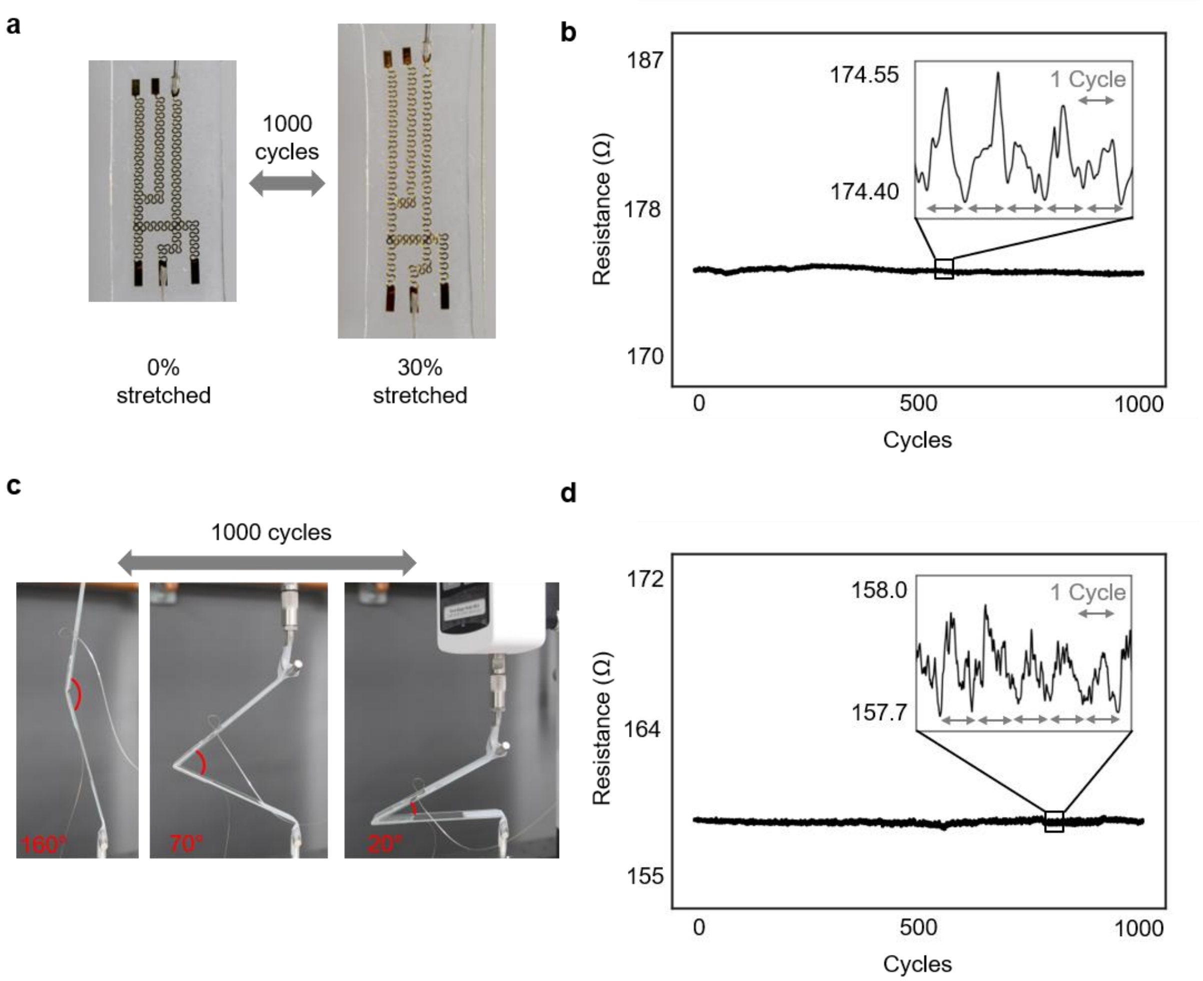

Figure S14. Cyclic stretching and bending of the stretchable connector. (a, c) Photos showing the set up for the cyclic (a) stretching and (b) bending tests. The resistance was measured by connecting two flexible film cables to the connector while the actuations were enabled by the motorized test stand. (b, d) Resistance measurements of the (b) stretching and (d) bending tests. The average variation in the resistance values were approximately 0.2% (0.5 **Ω**) and 0.4% (0.7 **Ω**) for cyclic stretching and bending, respectively. The zoom-in insets show detail data with each arrow representing one cycle.

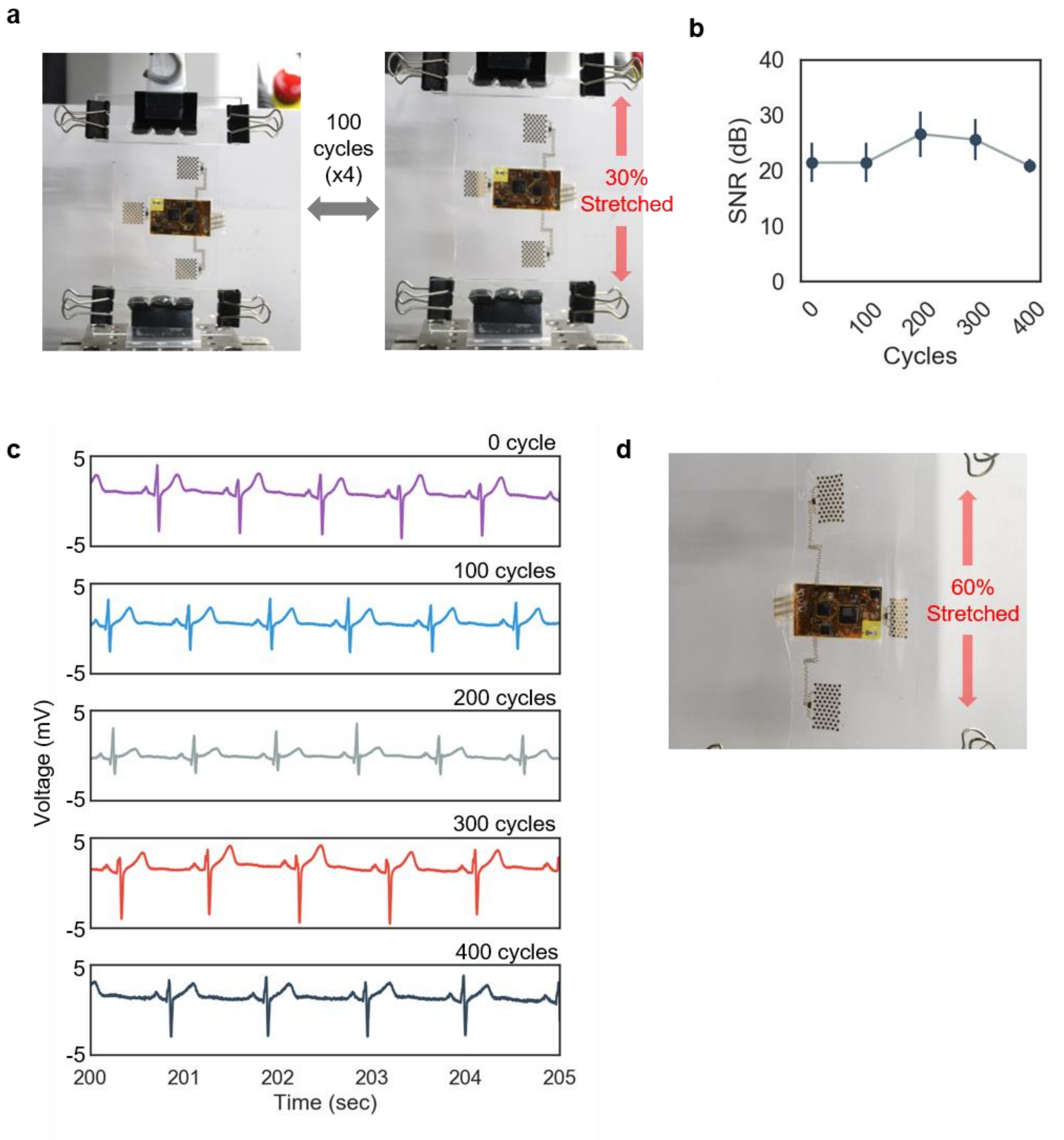

Figure S15. Cyclic stretching of SHE. (a) Experimental set up for cyclic stretching of SHE. ECG was measured before stretching and after every 100 cycles up to 400 total cycles. (b) SNRs of the ECGs measured before and after each 100 cycle. (c) 5-second window of ECG waveform throughout the test. (d) Maximum stretchability test for SHE.

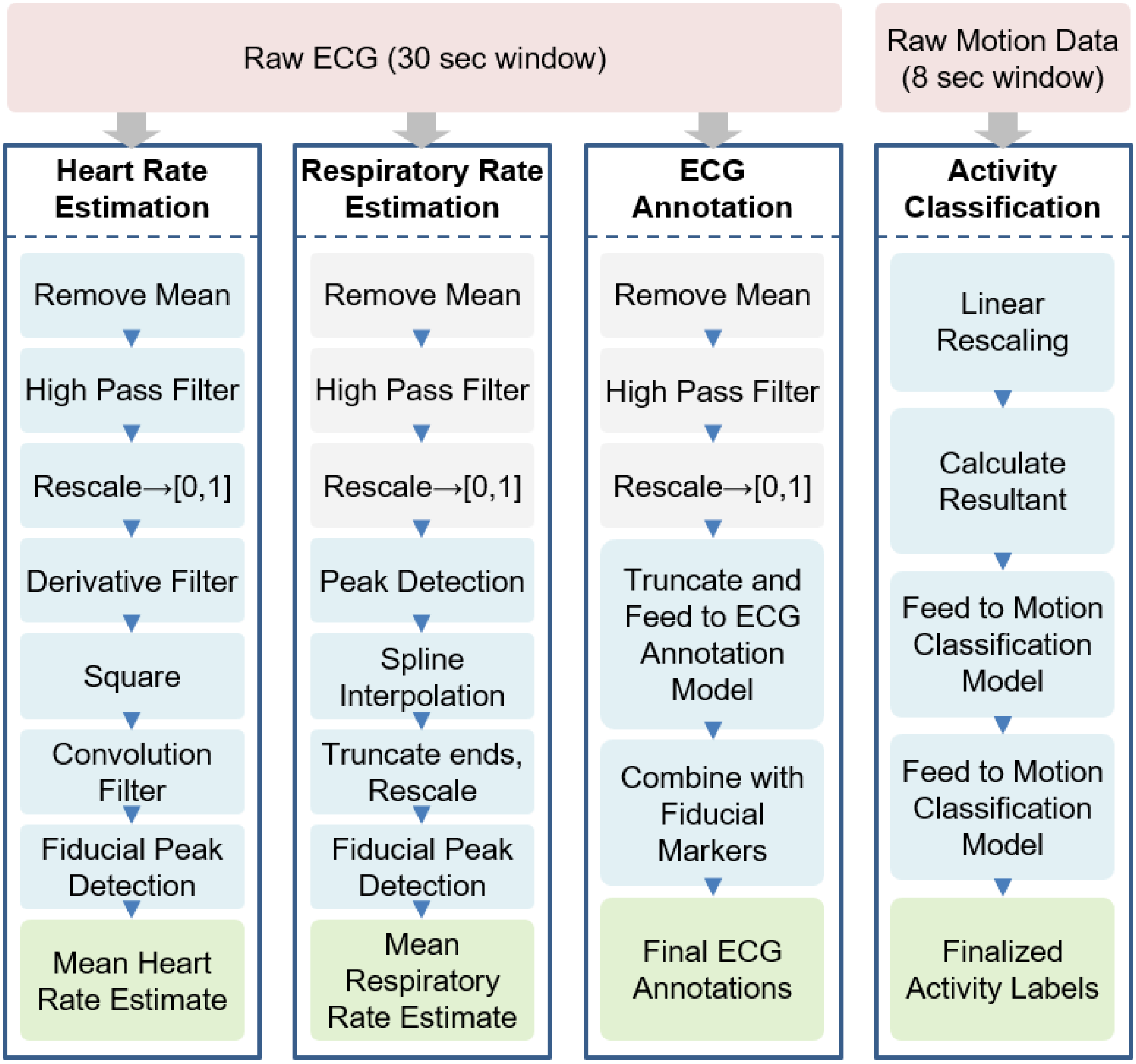

Figure S16. Derivation flowcharts. Detail description showing the derivation of heart rate, respiratory rate, ECG annotation, and activity classification. Note that some preprocessing steps are shared for HR, RR estimation and ECG annotation, and is therefore only performed once in the mobile implementation.

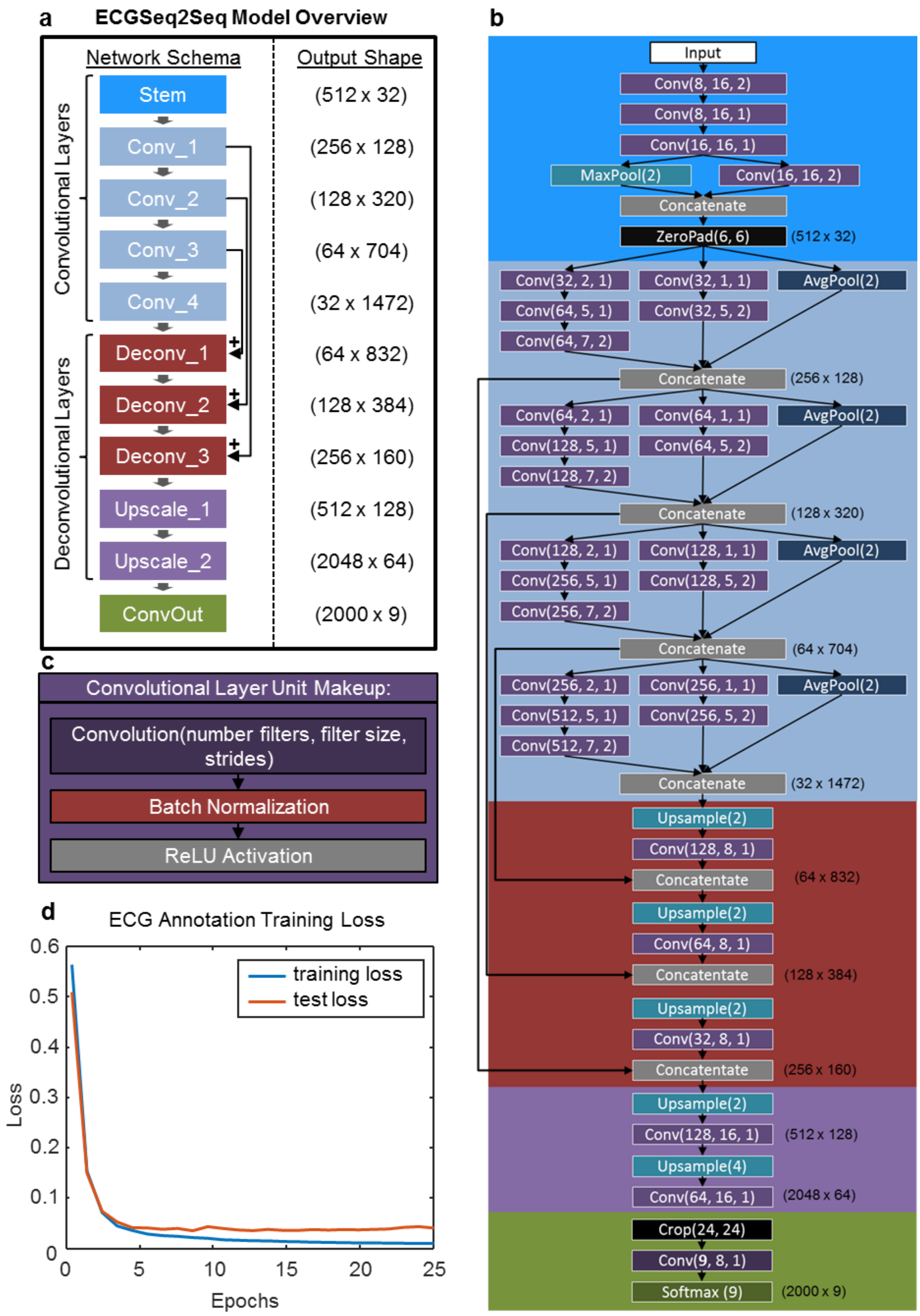

Figure S17. Details of the ECG classification algorithm. (a) High level network schema of ECGSeq2Seq model, (b) ECGSeq2Seq model with all components shown including residual connections. (c) Makeup of each purple convolution unit shown in (b). (d) Training and test loss vs epochs during training process.

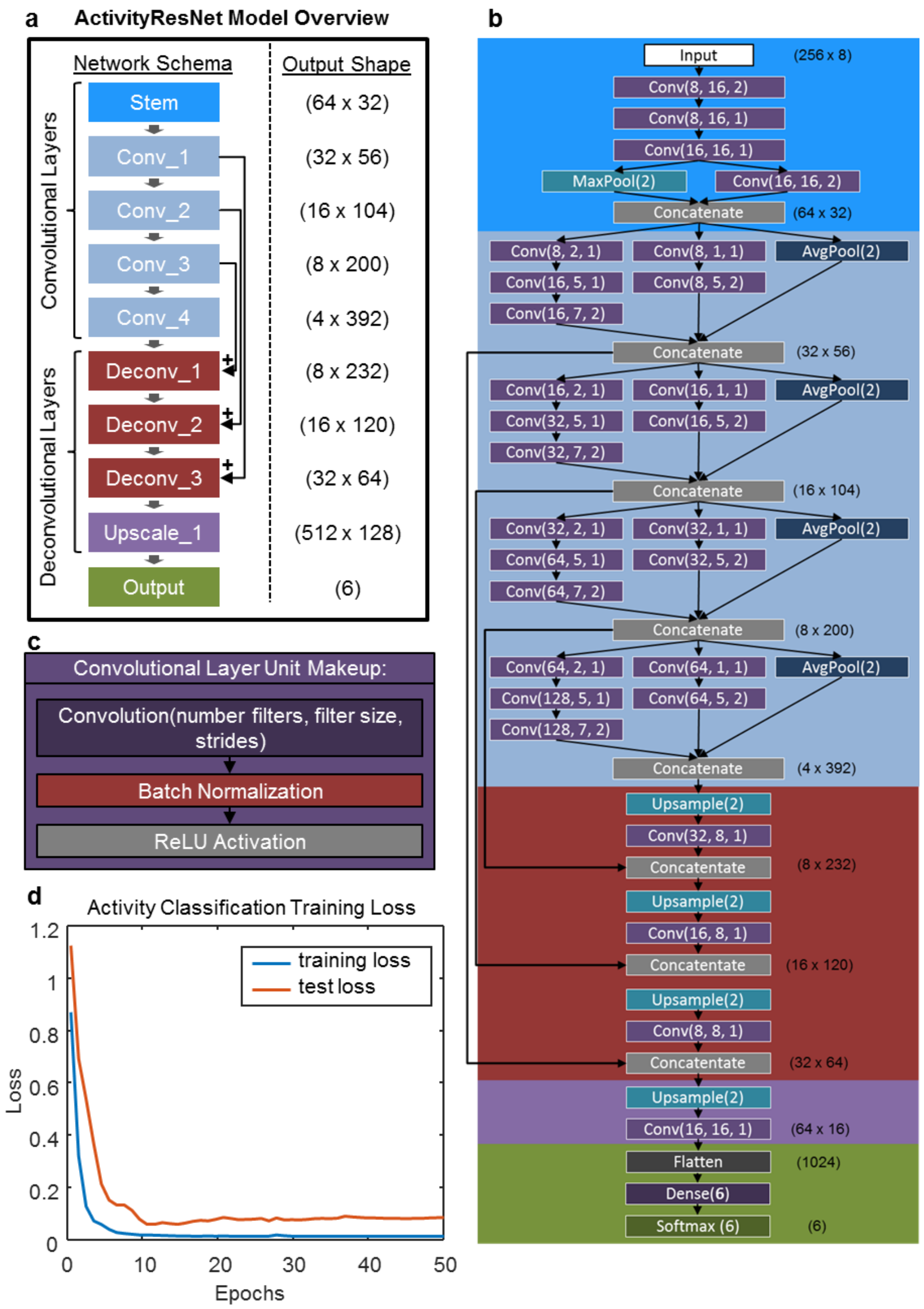

Figure S18. Details of the activity classification algorithm. (a) High level network schema of the ActivityResNet model. (b) ActivityResNet model with all components shown, including residual connections. (c) Makeup of each convolutional unit shown in (b). (d) Training and test loss vs epochs during training process.

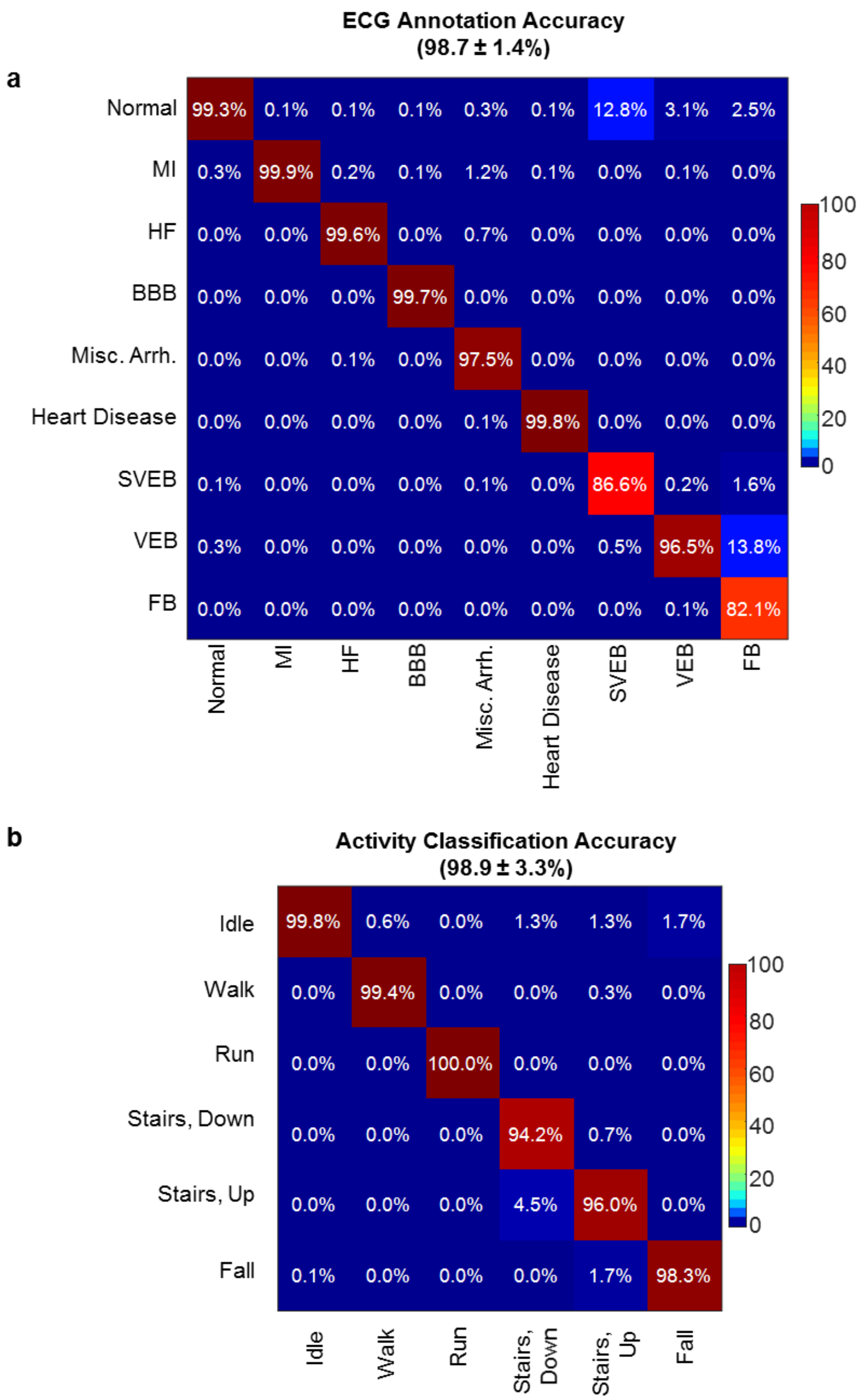

Figure S19. ECG and Activity Classification Confusion Matrices. (a) Confusion matrix for the 9-class ECGSeq2Seq semantic segmentation model. (b) Confusion matrix for the 6-class ActivityResNet classification model.

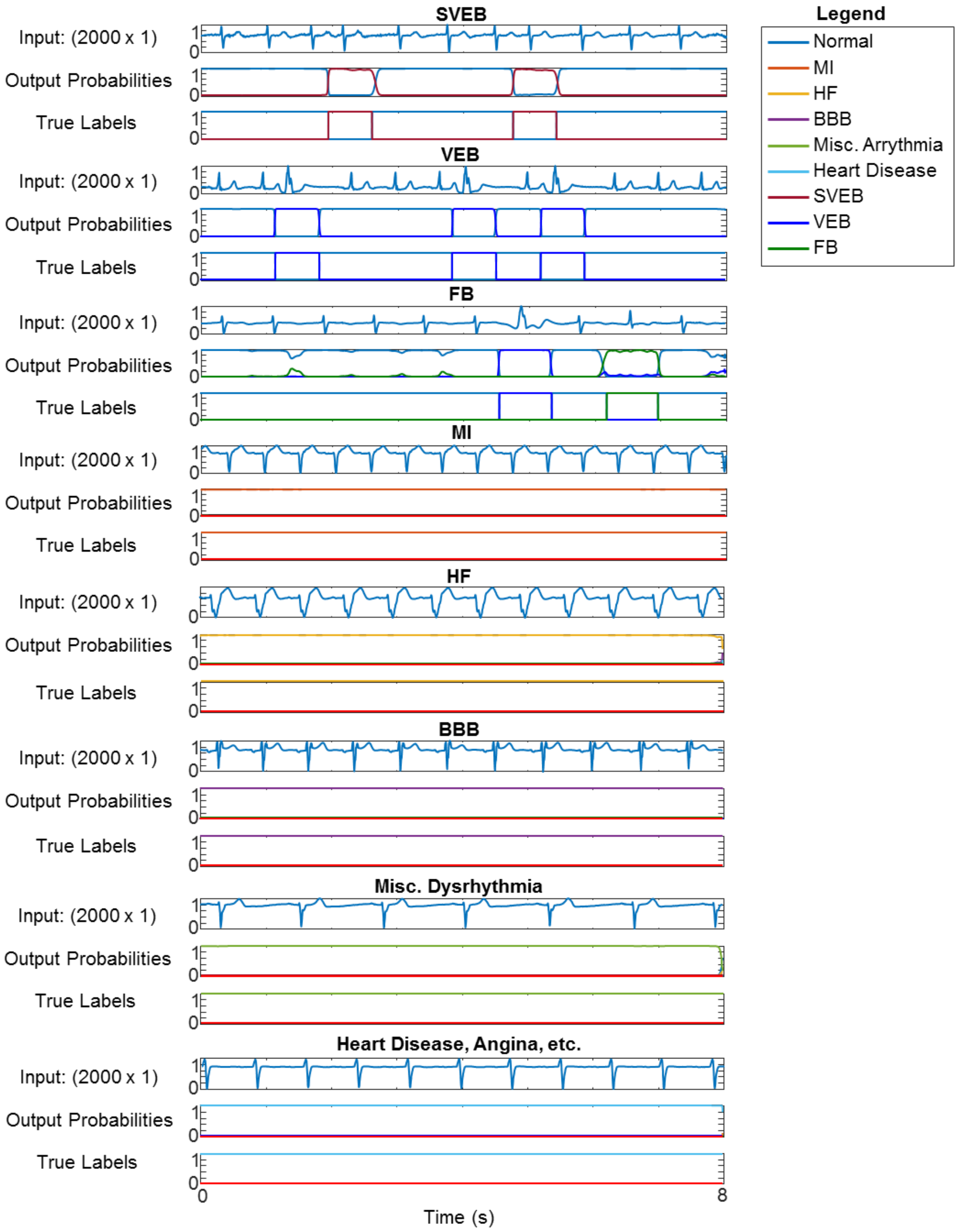

Figure S20. ECG Semantic Segmentation Examples. Samples from each of the classes shown and labeled above, with individual beats labeled, or entire rhythms labeled.

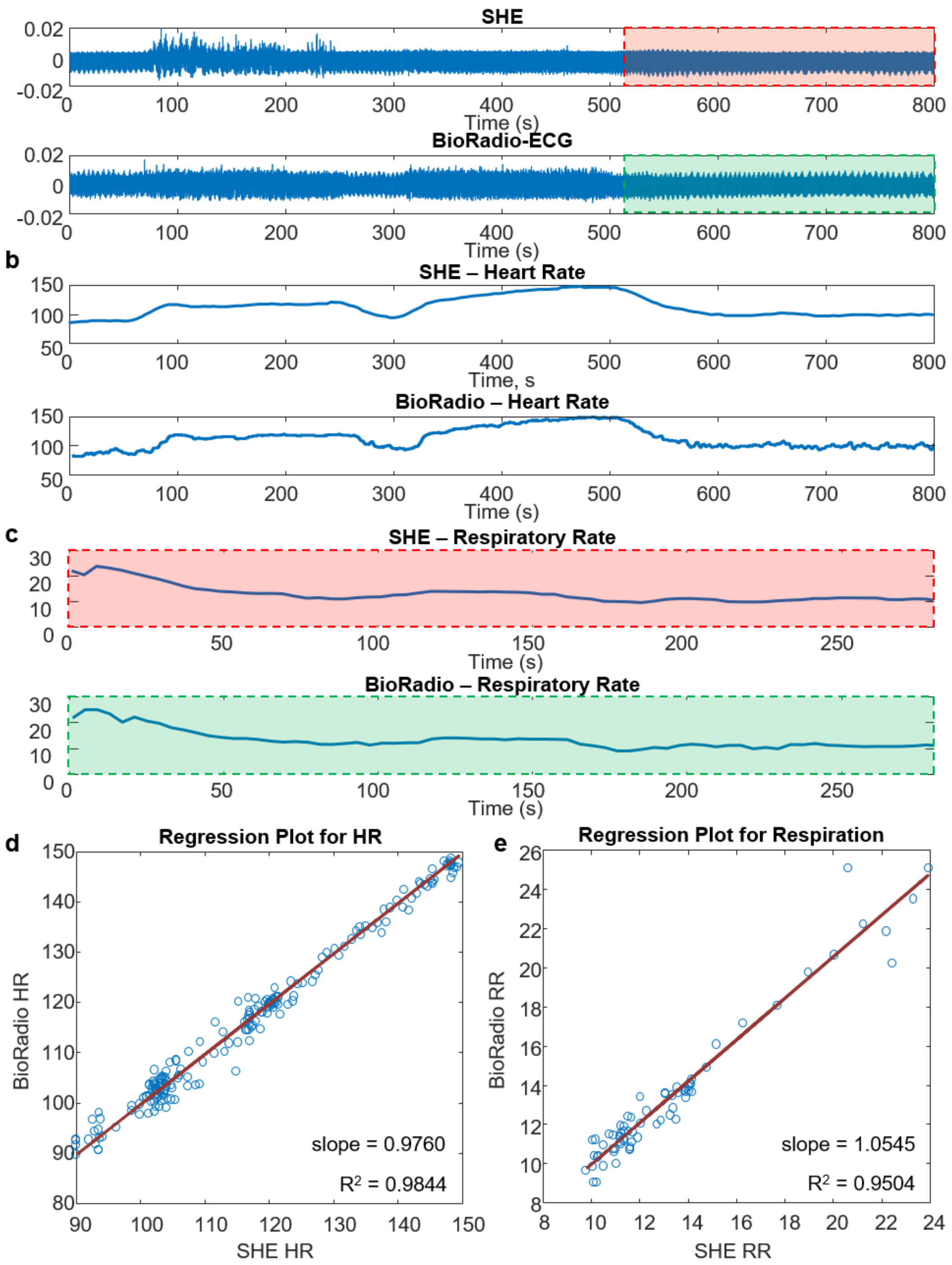

Figure S21. HR and RR Validation with Commercial Device. (a) Comparison of raw ECG waveforms for SHE (top) and BioRadio (bottom). (b) Comparison of derived SHE heart rate (top) versus BioRadio heart rate (bottom). (c) Comparison of respiratory rate for SHE (top) versus BioRadio (bottom). Note that this is recorded from the latter cool-down portion of the original recorded, as color coded in (a). (d) Regression plot for heart rate between SHE and commercial BioRadio, with corresponding slope and R^2 values. (e) Regression plot for respiratory rate between SHE and BioRadio, with corresponding slope and R^2 values.

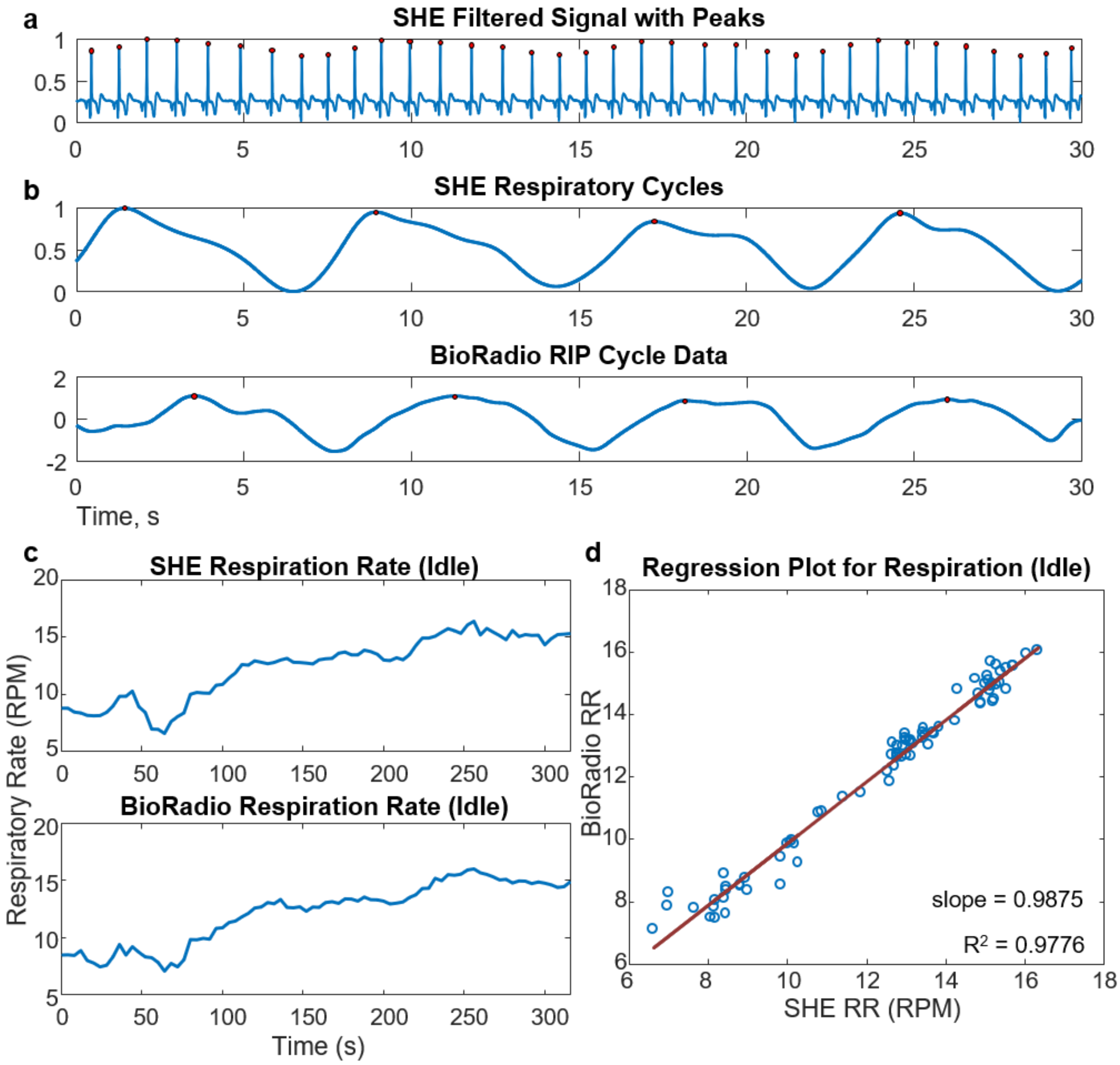

Figure S22. Validation of RR Derivation with Commercial Device. (a) Filtered ECG with fiducial markers used for respiratory rate derivation. (b) Derived respiration cycles from SHE (top) versus BioRadio respiratory inductance plethysmography (RIP) cycles (bottom). (c) Idle respiration rates over time for SHE (top) and BioRadio (bottom). (d) Regression plot for idle respiration between SHE and BioRadio, with corresponding slope and R^2 values.

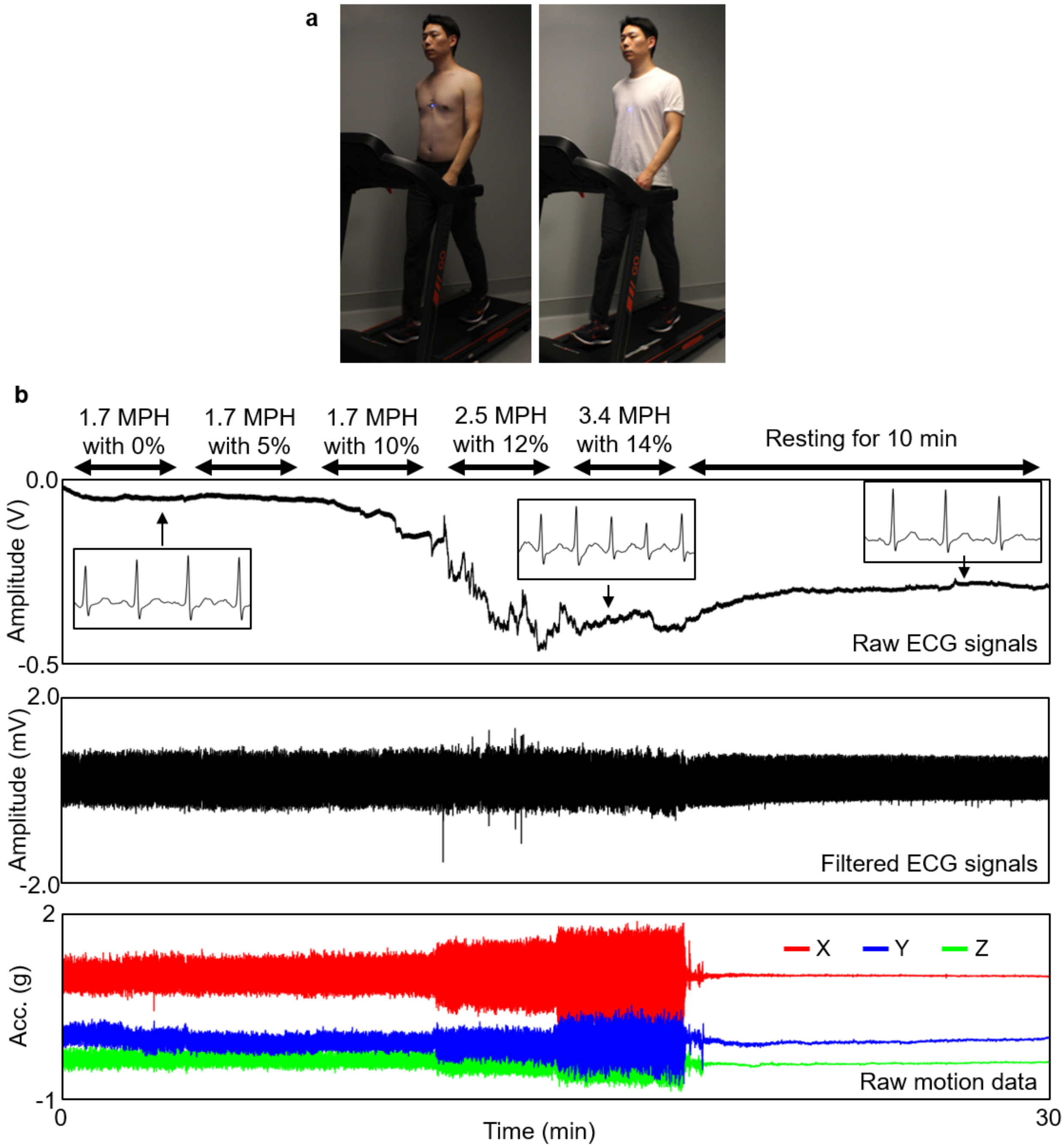

Figure S23. Using SHE for a modified Bruce protocol. (a) Photos show a user performing a treadmill exercise while wearing a SHE. (b) Raw and filtered ECG acquired by SHE from the 5-stage Bruce protocol followed by a 10-minute resting.

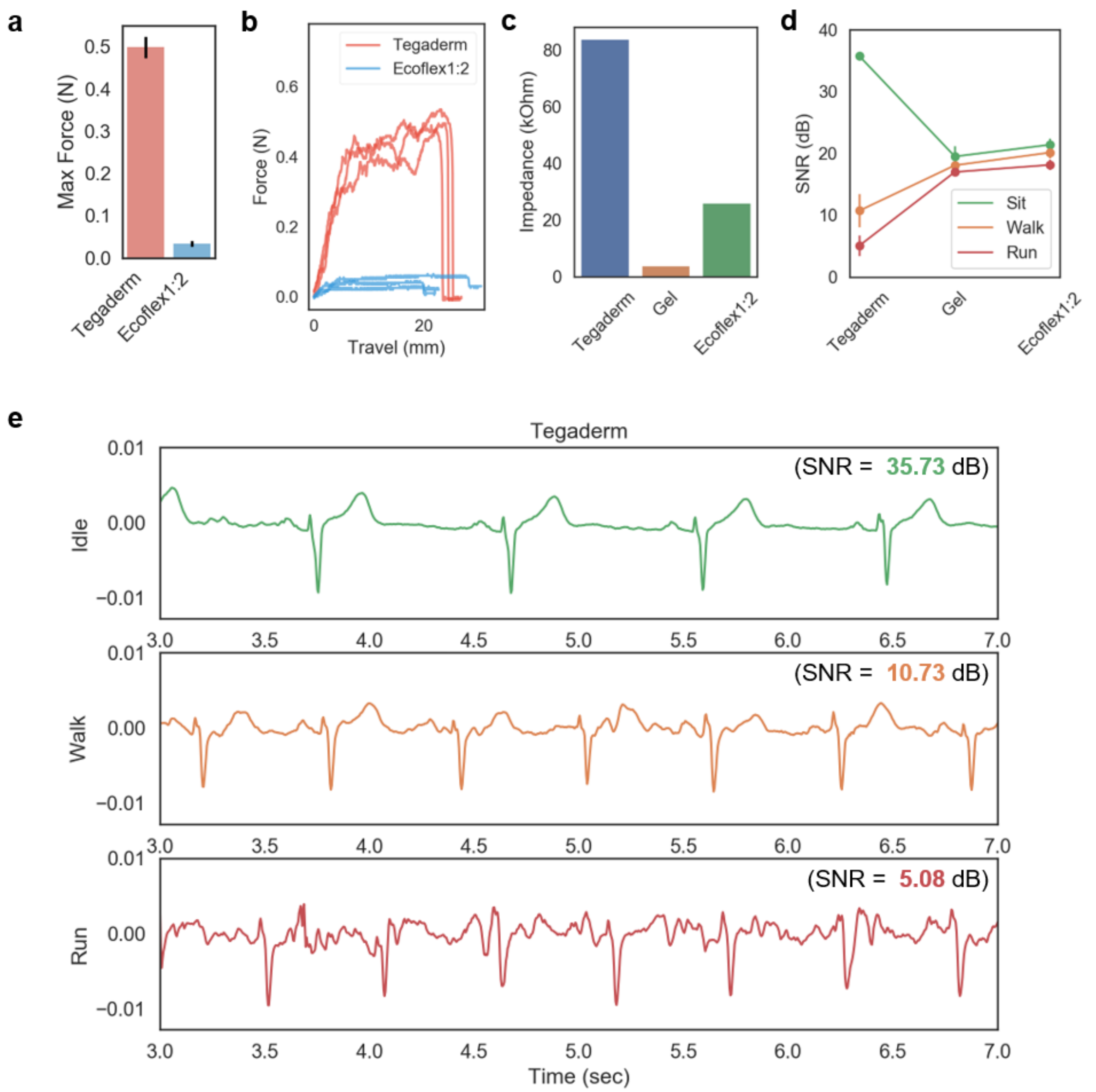

Figure S24. Comparison of substrate materials and properties of Tegaderm-based SHE. (a) Average maximum forces measured from peeling 1 cm x 2 cm sample substrates from the forearm. (b) Force vs. displacement plots measured from Tegaderm and Ecoflex1:2 samples. (c) Skin-electrode impedance comparison. (d) SNRs of ECG measured with Tegaderm-, gel-, and Ecoflex1:2-based electrodes. (e) Changes in the ECG waveform measured with Tegaderm-based electrodes.

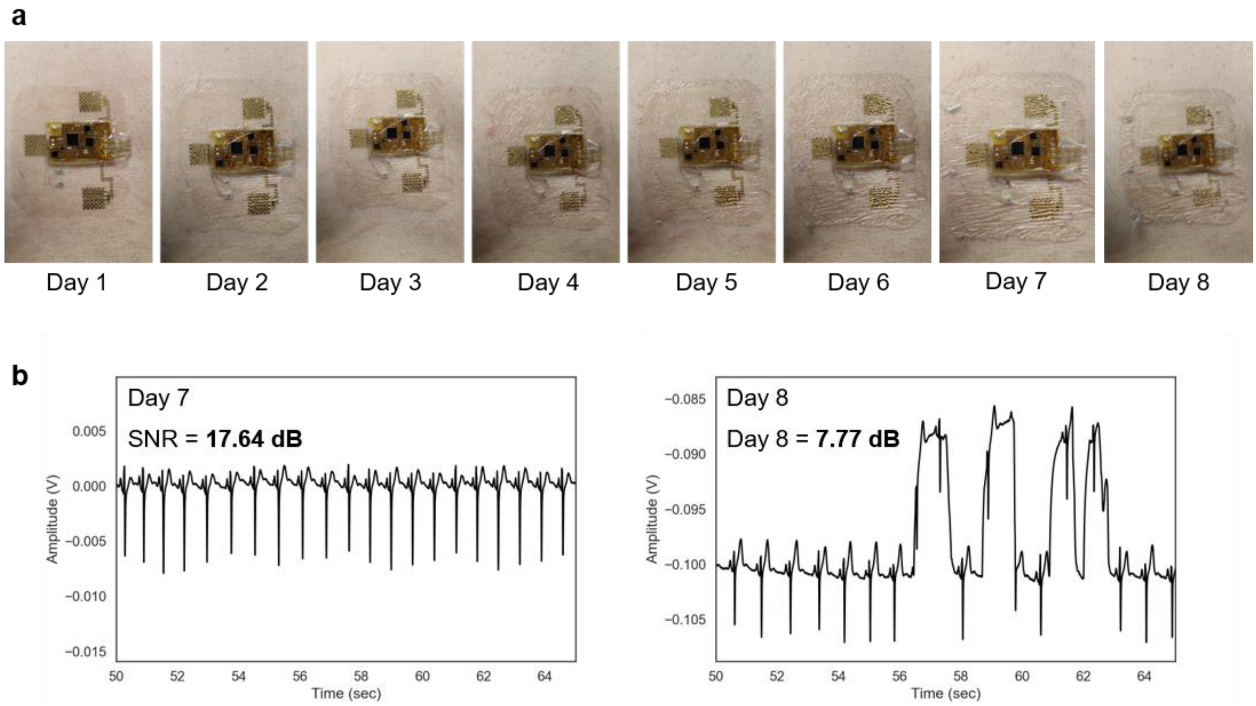

Figure S25. Prolonged application of SHE. (a) Photos capturing the progression of the Tegaderm-integrated SHE condition during the prolonged (8 days) application. (b) Significant decrease in SNR between Day 7 and Day 8, where poor electrode contact results in the fluctuation in the ECG waveform.

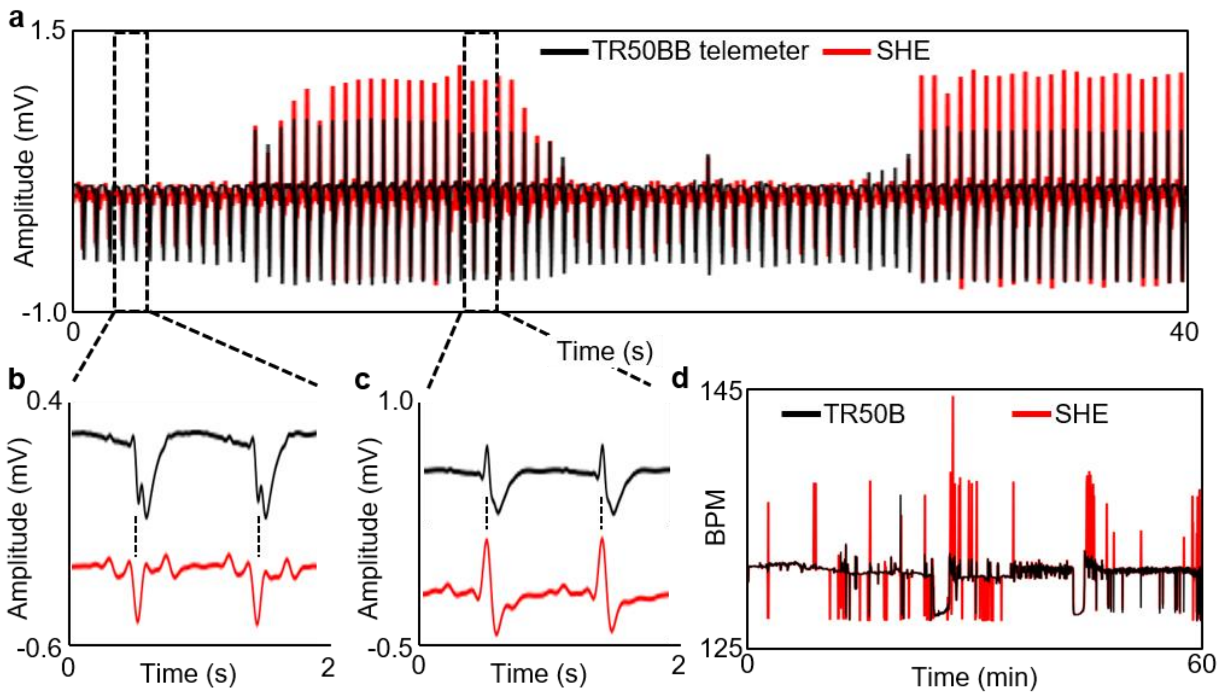

Figure S26. Detail comparison between TR50BB and SHE. (a) Overlay of ECG collected simultaneously by TR50BB and SHE. (b-c) Close inspection of the two waveforms. (d) Comparison of BPM data analyzed from the two ECG data.

Table S1. List of surface mount components used in SHE. The table describes the component symbol (to match the symbols used in Fig. S2), description, value, and part number.

Component	Description	Value	Part number
U1	3.3 voltage regulator	N/A	TPS63001
U2	Analog front-end	N/A	ADS1292
U3	Bluetooth PSoC	N/A	NRF52832-QFAA-R
U4	Motion sensor	N/A	MPU-9250
U5	Current limit active-low load switch	N/A	TPS22941
L1	0402 inductor	2.2 µH	N/A
L3	0402 inductor	15 nH	N/A
L4	0603 inductor	10 µH	N/A
L5	0402 inductor	10 nH	N/A
L6	0402 inductor	2.7 nH	N/A
C1, C10, C14, C15	0402 ceramic capacitor	10 µF	N/A
C2	0402 ceramic capacitor	22 µF	N/A
C3, C5	0402 ceramic capacitor	4.7 nF	N/A
C4	0402 ceramic capacitor	1.0 nF	N/A
C6, C7, C12, C22, C26, C33, C35, C36	0402 ceramic capacitor	0.1 µF	N/A
C8, C11, C13	0402 ceramic capacitor	1.0 µF	N/A
C9	0402 tantalum capacitor	1.0 µF	N/A
C21	0402 ceramic capacitor	4.7 µF	N/A
C23	0603 ceramic capacitor	10 µF	N/A
C24, C25, C28, C29	0402 ceramic capacitor	12 pF	N/A
C31	0402 capacitor	0.4 pF	N/A
R1, R2, R3, R4	0402 resistor	30 kΩ	N/A
R5, R6, R7, R8	0402 resistor	1 MΩ	N/A
R11. R12	0402 resistor	10 kΩ	N/A
A1	2.45 GHz RF chip antenna	N/A	2450AT18A100
F1	2.45 GHz low pass filter	N/A	2450FM07A0029
X1	32 MHz crystal	N/A	ECS-320-8-37CKM
X2	32.768 kHz crystal	N/A	ECS-.327-9-12-TR

Table S2. Summary of calculated Δγ values. The table lists all Δγ (N/m) values determined by the cylinder length, cylinder weight, and angles of the incline.

Angle (°)	Part 1	Part 2	Part 3	Part 4	Part 5
5	1.491	0.436	0.347	1.429	0.258
10	2.971	0.870	0.691	2.848	0.514
20	5.851	1.713	1.361	5.609	1.013
30	8.554	2.504	1.990	8.201	1.481

Movie S1. Demonstration of BLE connectivity. Movie verifies the consistent BLE connectivity between SHE and a tablet for distance exceeding 15 m.

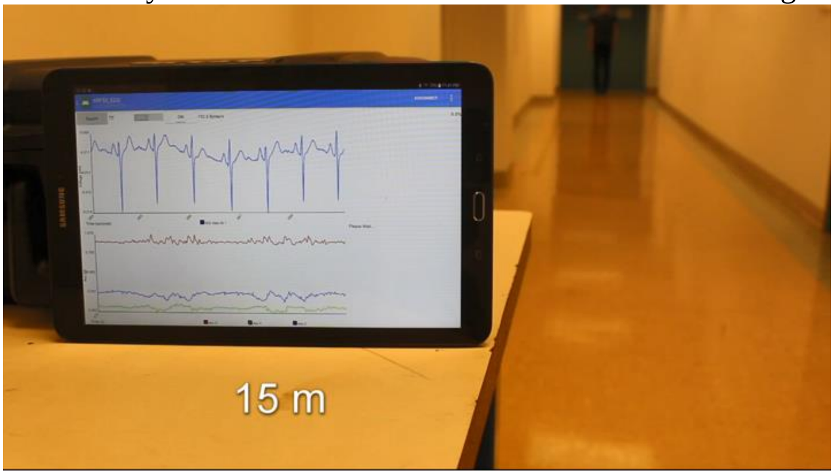

Movie S2. Cyclic stress test of SHE. Movie shows the experimental set up for the cyclic stress test.

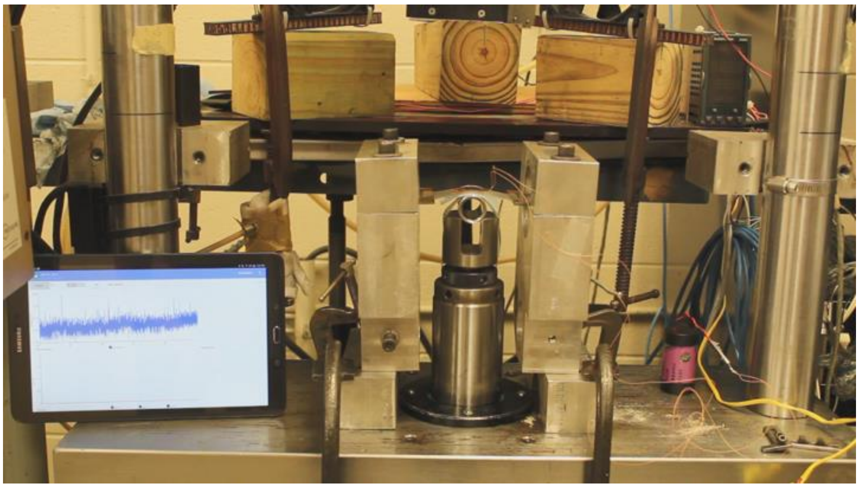

Movie S3. Real-time monitoring with SHE. Movie shows the real-time analysis of a healthy subject wearing the SHE.

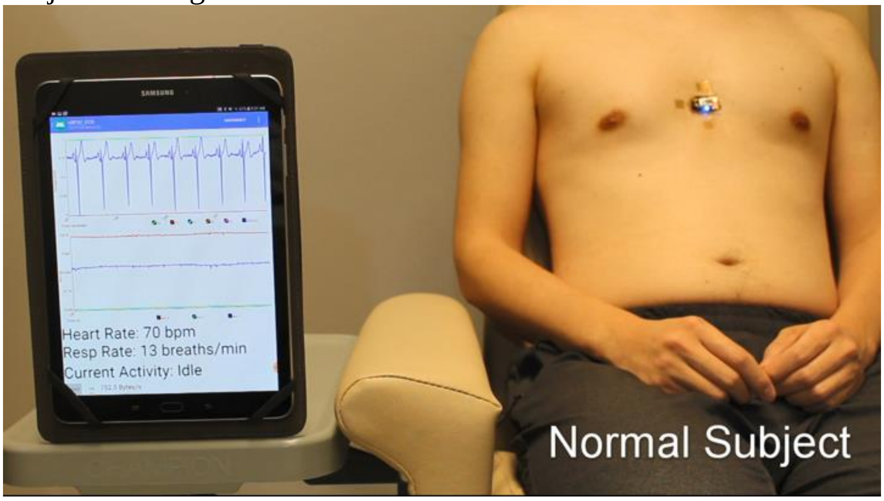

Movie S4. Real-time detection of ECG abnormality. Movie shows the Android application detecting the VEB in real-time.

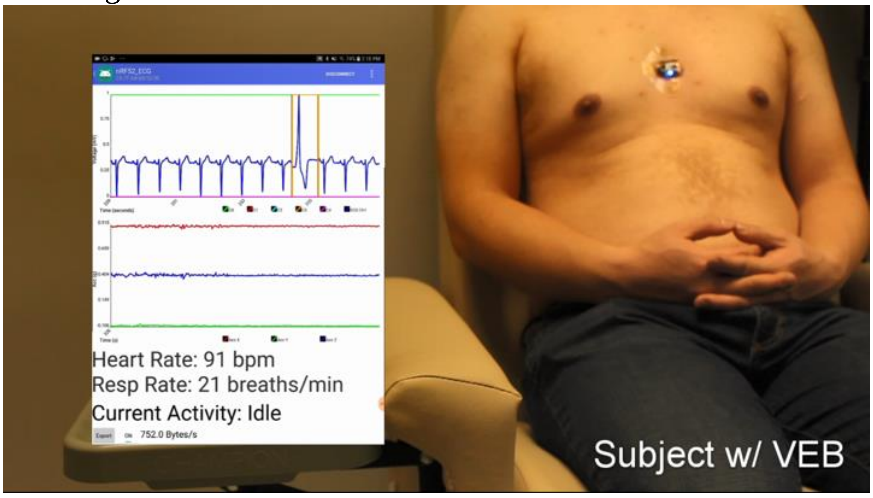

Movie S5. Real-time analysis of an active user. Demonstration of the real-time monitoring of the ECG, HR, RR, and activity using the Android application, which can be seen to correctly classify the 5 activity classes (idle, walk, stairs up, stairs down, and fall).

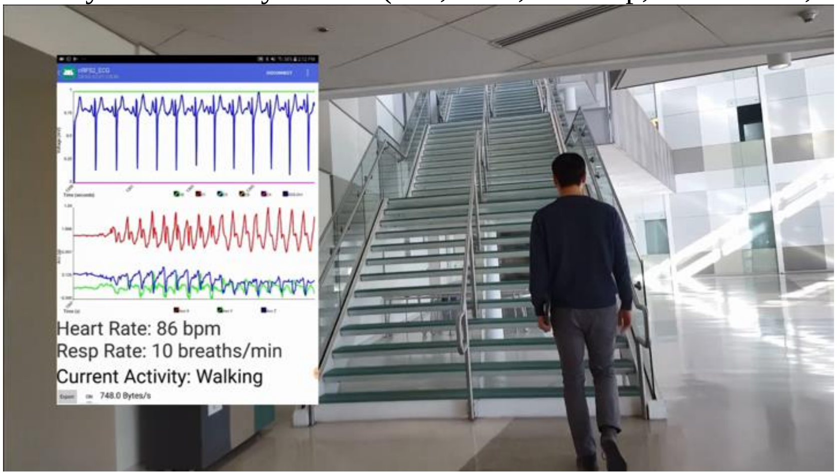

Movie S6. Application of SHE for treadmill exercises. Demonstration of SHE functionality as a multifunctional monitor during an exercise, such as the shown treadmill protocol.

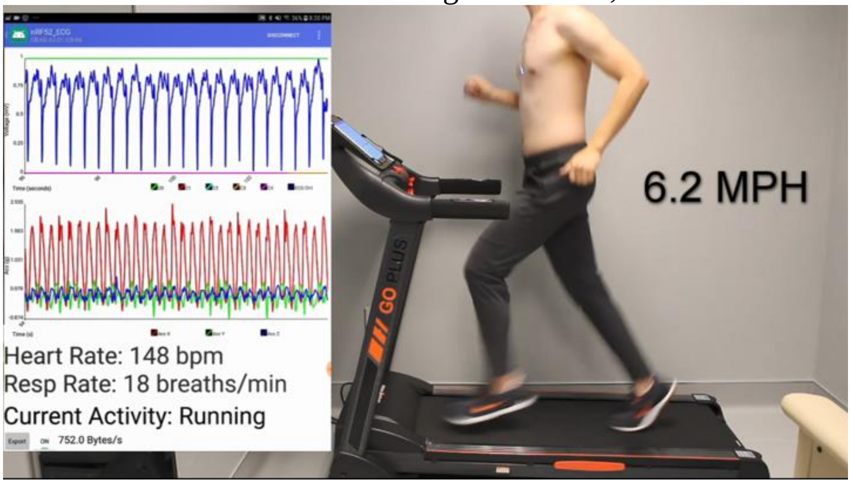

Movie S7. Tegaderm-integrated SHE as an animal research tool. Tegaderm-integrated SHE can be applied to small animal subjects for ambulatory cardiac monitoring.

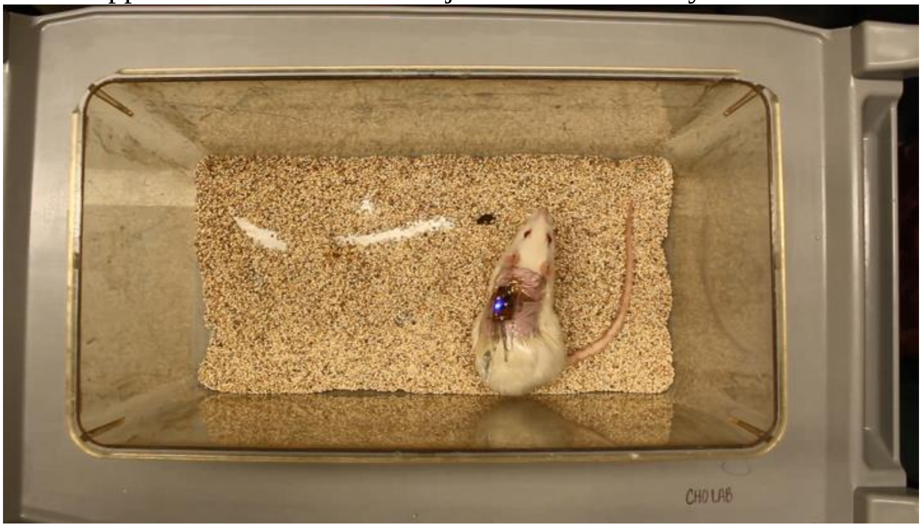

Movie S8. Comparison of ECG between TR50BB and SHE. The movie captures the simultaneous acquisition of the cardiac activity using TR50BB and SHE.

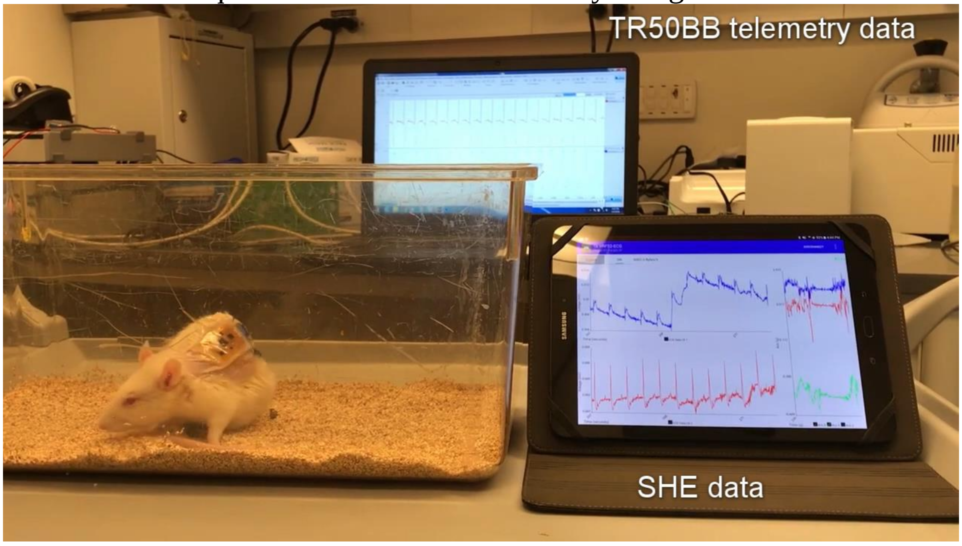

Supplement: Supplementary file 1 — Supplementary [file ADVS-6-1900939-s002.pdf]
